# Oxidative C-H/C-H Coupling of Dipyrromethanes with Azines by TiO_2_-Based Photocatalytic System. Synthesis of New BODIPY Dyes and Their Photophysical and Electrochemical Properties

**DOI:** 10.3390/molecules26185549

**Published:** 2021-09-13

**Authors:** Maria A. Trestsova, Irina A. Utepova, Oleg N. Chupakhin, Maksim V. Semenov, Dmitry N. Pevtsov, Lyubov M. Nikolenko, Sergey A. Tovstun, Anna V. Gadomska, Alexander V. Shchepochkin, Gregory A. Kim, Vladimir F. Razumov, Irina B. Dorosheva, Andrey A. Rempel

**Affiliations:** 1 Department of Organic and Biomolecular Chemistry, Ural Federal University, 19 Mira Street, 620002 Ekaterinburg, Russia; maria.trestcova@urfu.ru (M.A.T.); chupakhin@ios.uran.ru (O.N.C.); semmaximwork@gmail.com (M.V.S.); i.b.dorosheva@urfu.ru (I.B.D.); rempel.imet@mail.ru (A.A.R.); 2 Institute of Organic Synthesis of the Russian Academy of Sciences, 22 S. Kovalevskoy Street, 620108 Ekaterinburg, Russia; sasha-mmm@mail.ru (A.V.S.); kim-g@ios.uran.ru (G.A.K.); 3 Moscow Institute of Physics and Technology, 9 Institutsky Lane, 141701 Dolgoprudny, Russia; pevtsovdm@gmail.com (D.N.P.); tovstun@icp.ac.ru (S.A.T.); razumov@icp.ac.ru (V.F.R.); 4 Institute of Problems of Chemical Physics of the Russian Academy of Sciences, 1 Academician Semenov Avenue, 142432 Chernogolovka, Russia; nav@icp.ac.ru (L.M.N.); ann.gadomsky@gmail.com (A.V.G.); 5 Institute of Metallurgy of the Ural Branch of the Russian Academy of Sciences, 101 Amundsena Street, 620016 Ekaterinburg, Russia

**Keywords:** dipyrromethane, BODIPY, azine, TiO_2_, C-H/C-H couplings, ONSH, photocatalysis

## Abstract

Oxidative C-H/C-H coupling reactions of dipyrromethanes with azines in the presence of a heterophase oxidative photocatalytic system (O_2_/TiO_2_/visible light irradiation) were carried out. As a result of cyclization of obtained compounds with boron trifluoride etherate, new hetaryl-containing derivatives of 4,4-difluoro-4-boron-3*a*,4*a*-diaza-*s*-indacene were synthesized. For the obtained compounds, absorption and luminescence spectra, quantum yields of luminescence as well as cyclic volt-amperograms were measured.

## 1. Introduction

**Figure 1:**
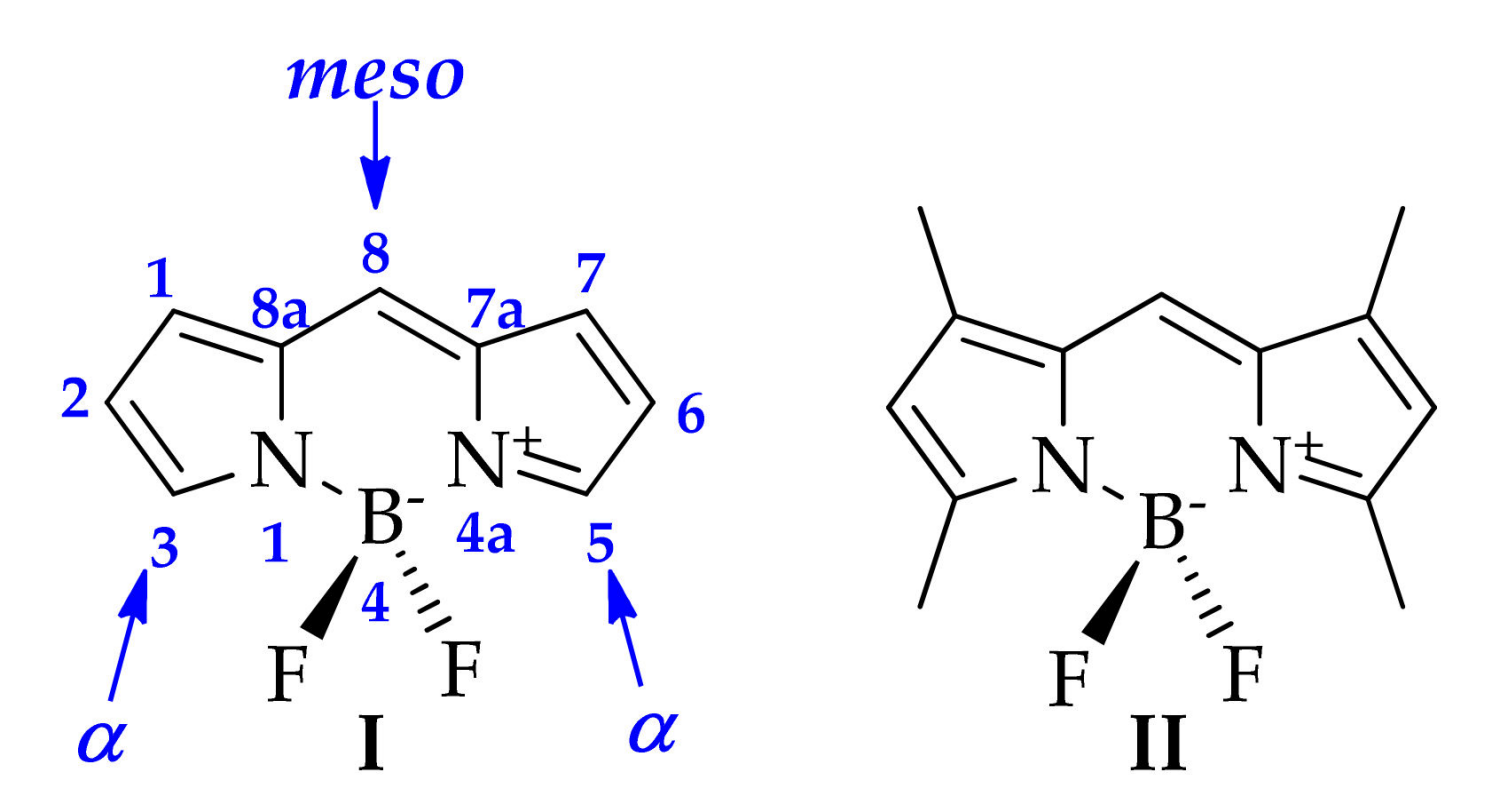
4,4-difluoro-4-boron-3*a*,4*a*-diaza-*s*-indacene (**I**), 4,4-difluoro-1,3,5,7-tetramethyl-4-boron-3a,4a-diaza-s-indacene (**II**).

4,4-Difluoro-4-boron-3*a*,4*a*-diaza-*s*-indacene derivatives (**I**, Figure 1) represent an important class of organic luminophores. The first publication on the synthesis of some derivatives of the presented series appeared in 1968 [1]. Further, it was shown that various 1,2,3- and 5,6,7-methyl- and ethyl-substituted derivatives of compound **I** have high quantum yields of luminescence in various solvents [2].

It was later found that derivatives of compound **I** can be useful for biological applications [3], as dyes for lasers [4], including as fluorescent labels in biology [5]. Currently, the name given to this class of compounds is BODIPY [6,7]. Most often, the term BODIPY refers to compound **I** [8,9,10,11,12] and sometimes to its 1,3,5,7-tetramethyl-substituted derivative (**II**, Figure 1) [6,13,14]. These dyes have similar optical properties. However, their *meso*-phenyl-substituted derivatives can have very different photoluminescence quantum yields because the methyl substituents at the positions 1 and 7 sterically block the rotation of the phenyl ring [15]. It should be noted that compound **I** was synthesized only in 2009 [8,9,16]. In derivative **I**, all atoms except for the fluorines lie almost in the same plane [8,9]. The optical properties of this compound are weakly dependent on solvent [8,17]. The introduction of various substituents into structure **I** allows varying these properties and make them dependent on the medium, which is in demand in various applications [10]. BODIPY derivatives are currently widely used as fluorescent dyes [18], chemosensors [19,20], fluorescent probes [21,22], laser dyes [23,24] and as compounds for photodynamic therapy [25,26,27].

BODIPY dyes with aromatic and heteroaromatic substituents, which are used for in vivo imaging, are promising for practical applications since they reduce the harmful effect of radiation on living systems, allow it to penetrate deeper into organic tissues and reduce autofluorescence of biological objects [28]. Therefore, new methods of obtaining and studying properties of various derivatives of BODIPY fluorophores are being actively developed [28,29,30].

Methods for the synthesis of heteroaryl derivatives of BODIPY in most cases are based on the one hand on multistep methods for the construction of heteroaryl-substituted pyrrole synthons [31,32] and on the other hand on the use of transition metal-catalyzed cross-coupling reactions of halogen derivatives of BODIPY with nitrogen-containing heterocycles [33,34,35,36,37]. These transformations require the use of metal-based catalysts and additional functionalization of starting reagents. In addition, a significant disadvantage is a mandatory additional purification of the target product. A more atom-economical method of incorporating functional groups into the BODIPY nucleus at positions 3,5 is an oxidative substitution of α-hydrogen by fragments of C-, N-nucleophiles [38]. In addition, examples of radical C-H arylation/heteroarylation of BODIPY dyes using aryldiazonium salts are known [39,40], ferrocene is used to generate aryl radical species in these transformations.

In our previous works, we proposed the oxidative C-H/C-H couplings of azines with aromatic and heteroaromatic nucleophiles, including pyrrole, in the presence of the heterogeneous air O_2_/TiO_2_ catalyst/light irradiation photocatalytic system [41,42,43]. The method allows one to selectively perform direct C-H functionalization of various substituted and unsubstituted mono-, di- and triazines as well as benzoannulated analogs.

This work reports for the first time a new synthetic technique for modifying dipyrromethanes as the main starting material for BODIPY dyes via direct oxidative C-H functionalization. It was found that dipyrromethanes undergo direct C-H/C-H coupling reactions with azines under aerobic conditions in the presence of a heterogeneous TiO_2_ photocatalyst. As a result of further cyclization of obtained compounds, new mono- and diazinyl-substituted BODIPY dyes were obtained, absorption and luminescence spectra, and quantum yields of luminescence in tetrachlorethylene and benzene, as well as cyclic voltamperograms in dichloromethane were measured.

## 2. Results

### 2.1. Synthesis

**Scheme 1:**
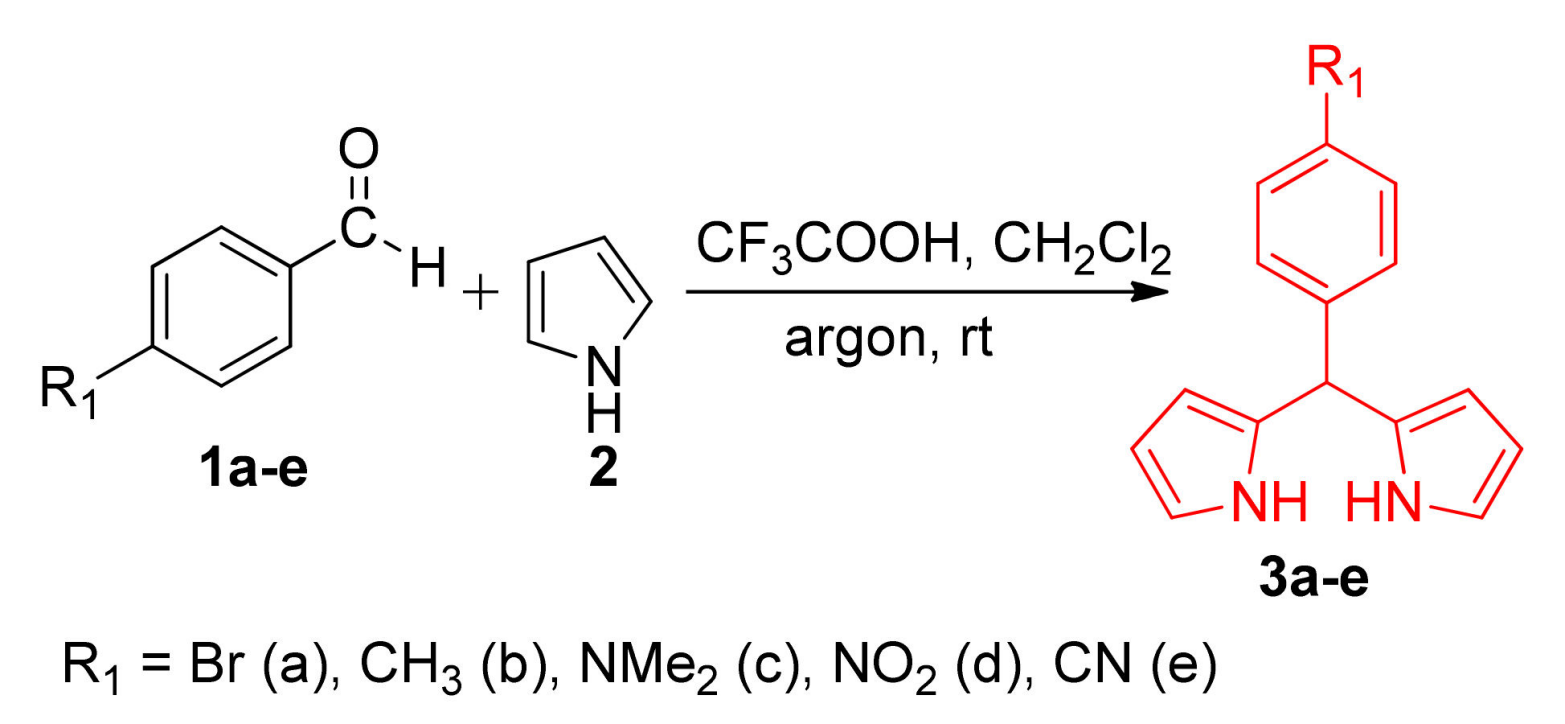
Synthesis of dipyrromethanes **3a**–**e**.

Starting dipyrromethanes **3a**–**e** were synthesized according to previously published procedures [44,45,46,47] by condensation of aldehydes **1a**–**e** with an excess of pyrrole **2** at room temperature upon activation with trifluoroacetic acid (Scheme 1). 

It was found that, similar to the interaction of azines with pyrroles and indoles [41,42,43], reactions of oxidative photocatalyzed C-H/C-H coupling of azines with dipyrromethanes under aerobic conditions can be successfully carried out. TiO_2_ obtained by a sol-gel method followed by annealing at 800 °C for 1 h in a hydrogen atmosphere was used as a photocatalyst. This catalyst was previously studied in the reaction of oxidative coupling of acridine with indole [48]. It was found that in its presence the reaction proceeds upon irradiation in the visible light range (Xe lamp, 5000 K, 35 W, using a yellow light filter, λ > 480 nm). Moreover, the synthesized TiO_2_ afforded the target product with a high yield compared to the commercially available TiO_2_ catalysts, Degussa P25 and Hombifine N. Optimization of the synthesis conditions was carried out using acridine **4a** having one reaction center and 5-(4-bromophenyl)dipyrromethane **3a** as an appropriate (simple and convenient) model (Table 1). Experiments were initially carried out in acetic acid upon irradiation with light in the visible range (EvoluChem™ LED, Beverly, MA, USA, 18 W, 425 nm) under aerobic conditions in the presence of nanostructured TiO_2_. It was found that as a result of the reaction at room temperature, monosubstituted dipyrromethane **5a** is formed in a yield of 23%, while under refluxing resinification of the reaction mass occurs, which is probably due to the thermal instability of these compounds in an acidic medium. Replacing acetic acid with trifluoroacetic acid or using boron trifluoride etherate as a Lewis acid did not lead to the desired result. In the course of further optimization, the reaction was carried out in dichloromethane with the addition of 20 equiv. of acetic acid in the presence of the oxidizing system air O_2_/TiO_2_ catalyst/light irradiation. It was found that, as a result, compound **5a** is formed at room temperature, and when the reaction mass is heated to 50 °C, disubstituted dipyrromethane **5b** is formed in 48% and 63% yields, respectively. Varying the reaction temperature from 30 °C to 45 °C resulted in a mixture of compounds **5a** and **5b**. Thus, the optimal conditions for carrying out oxidative C-H/C-H couplings of azines with dipyrromethanes is performing the reactions in dichloromethane in the presence of 20 equiv. of acetic acid.

We found that compounds **3a**–**e** react with azines **4a**–**c** under aerobic conditions in the presence of the TiO_2_ photocatalyst. In order to increase the activity of TiO_2_ and avoid coagulation of nanosized particles, the mixture of the starting compounds was sonicated for 5 min. The reactions of dipyrromethanes **3a**–**e** (1 equiv.) with acridine **4a** and 6-phenyl-[1,2,5]oxadiazolo[3,4-b]pyrazine **4b** (2 equiv.) were carried out in dichloromethane in the presence of 20 equiv. of acetic acid for 5 h (Table 2).

It was shown that 5-(4-bromophenyl)dipyrromethane **3a**, 5-(4-methylphenyl)dipyrromethane **3b** and 5-(4-N,N′-dimethylaminophenyl)dipyrromethane **3c** react with **4a** at room temperature to form monosubstituted dipyrromethanes **5a**,**d**,**f** (Table 2). The coupling reactions of 5-(4-nitrophenyl)dipyrromethane **3d** and 5-(4-cyanophenyl)dipyrromethane **3e** with **4a** under similar conditions lead to the formation of disubstitution products **5i**,**j**. When the temperature rises to 50 °C, dipyrromethanes **3a–c** react with acridine **4a** to form disubstitution products **5b**,**e**,**g**. Yields of compounds **5** range from 38% to 79%.

In turn, 6-phenyl-[1,2,5]oxadiazolo[3,4-b]pyrazine **4b** undergoes the coupling reactions with dipyrromethanes **3a,c** at room temperature with the formation of exclusively disubstituted products **5c,h** in 57% and 36% yields, respectively (Table 2). However, in the case of 5-(4-methylphenyl)dipyrromethane **3b**, 5-(4-nitrophenyl)dipyrromethane **3d** and 5-(4-cyanophenyl)dipyrromethane **3e**, the target products could not be obtained. It was found that dipyrromethanes **3a**–**e** easily react with acridine hydrochloride **4c** in *n*-BuOH at room temperature and with air bubbling for 5 h using the TiO_2_ photocatalyst to form compounds **5k**–**o** in 80–85% yields (Table 2).

Treatment of hydrochlorides **5k**–**o** with an aqueous solution of NaOH leads to the formation of products **5b**,**e**,**g**,**i**,**j**. The NMR spectra of compounds **5a–j** are available in Supplementary Materials. When using other azines (pyridine, pyrimidine, quinoxaline, quinazoline, 3,6-diphenyltriazine, quinoxalone) in the reaction under similar conditions, the target products could not be isolated.

BODIPY derivatives **6a**–**j** were prepared according to a standard synthesis procedure by oxidation of dipyrromethanes **5a**–**j** using 2,3-dichloro-5,6-dicyano-1,4-benzoquinone (DDQ) and subsequent cyclization with BF_3_·OEt_2_. For compounds **6a**,**d**–**i**, triethylamine was used as a base, and in the case of **6b**,**c**,**j**, diisopropylethylamine (DIPEA) was used. All reactions were carried out under inert conditions, yields of products **6** were 32–70% (Table 3). The NMR spectra for the synthesized BODIPY dyes **6a–j** are shown in Supplementary Materials.

Similar BODIPY derivatives without α-substituents were also synthesized: 4,4-difluoro-8-(4-*R*-phenyl)-4-boron-3*a*,4*a*-diaza-*s*-indacenes, where R = NMe_2_ (**7a**), Me (**7b**), Br (**7c**), CN (**7d**), NO_2_ (**7e**).

### 2.2. Photophysical Properties

**Figure 2:**
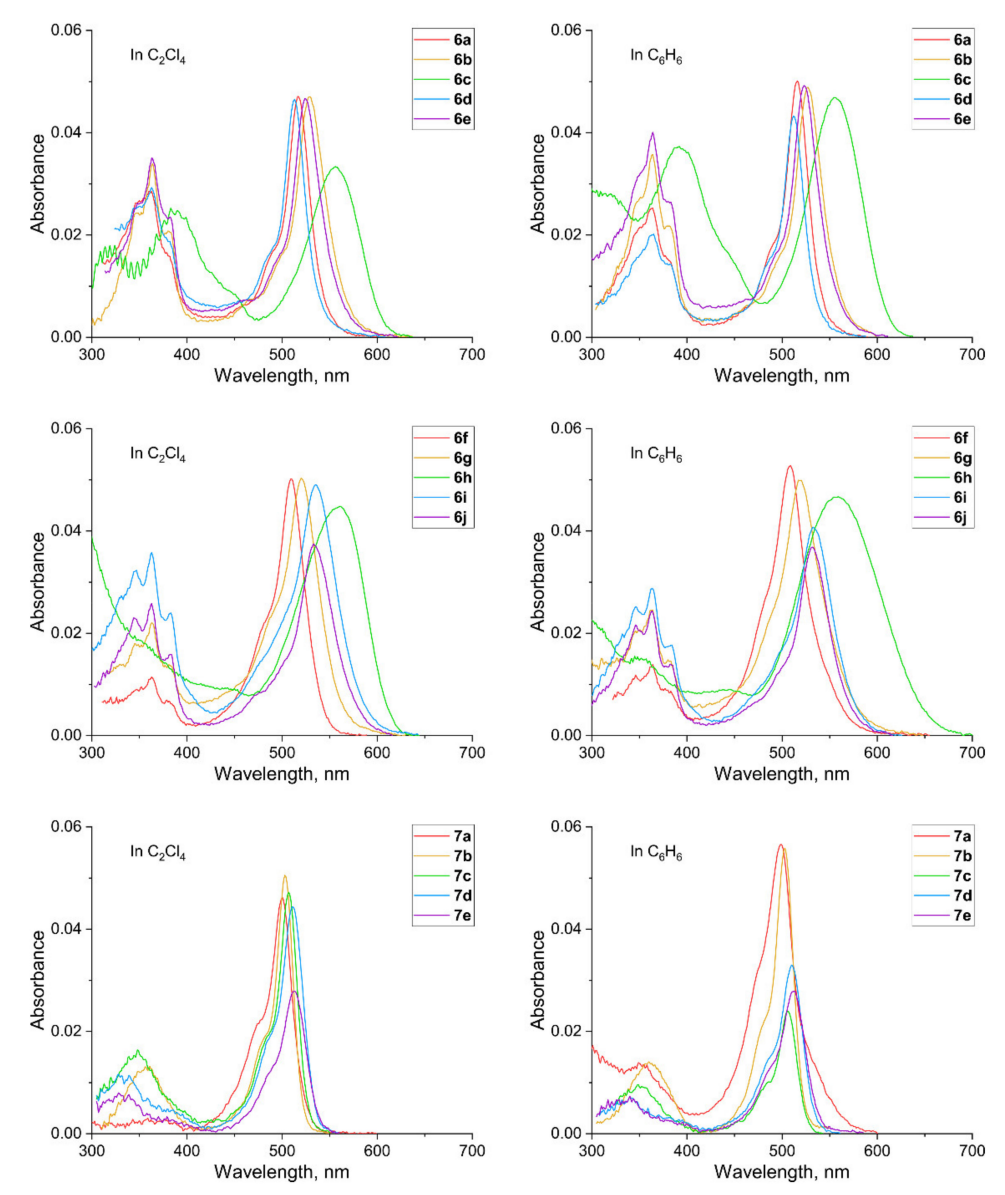
The absorption spectra of compounds **6a**–**j**, **7a**–**e**.

For all synthesized dyes, absorption and luminescence spectra were recorded, and also electrochemical measurements were carried out. The absorption spectra of the synthesized dyes are shown in Figure 2 and spectral-luminescent properties are given in Table 4.

As can be seen from Table 4, introduction of azinyl substituents at the positions 3 and 5 of the *meso*-aryl substituted BODIPYs leads in most cases to a red shift of the absorption and emission spectra and to an increase in the photoluminescence quantum yield. The effect is most pronounced when furazanopyrazine substituents are introduced into **7c**, leading to **6c**: the photoluminescence quantum yield in benzene increases from 0.036 to 0.81, while the peak of the emission spectrum shifts by 94 nm. This correlation holds for all BODIPY in this study except those having dimethylamino group because their properties are affected by photoinduced electron transfer from the phenyl *meso*-substituent to the indacene framework of the molecule. For all dyes having a dimethylamino group there is a strong dependence of the luminescent properties on the solvent. A small Stokes shift and a relatively high quantum yield are observed in nonpolar tetrachlorethylene, which indicates locally excited luminescence. However, when going to only slightly more polar benzene, the quantum yield drops sharply and the Stokes shift increases. For the dyes synthesized here without the dimethylamino group, the luminescence observed in tetrachlorethylene and benzene is locally excited and does not show features of the photoinduced charge transfer.

### 2.3. Electrochemical Properties

**Figure 3:**
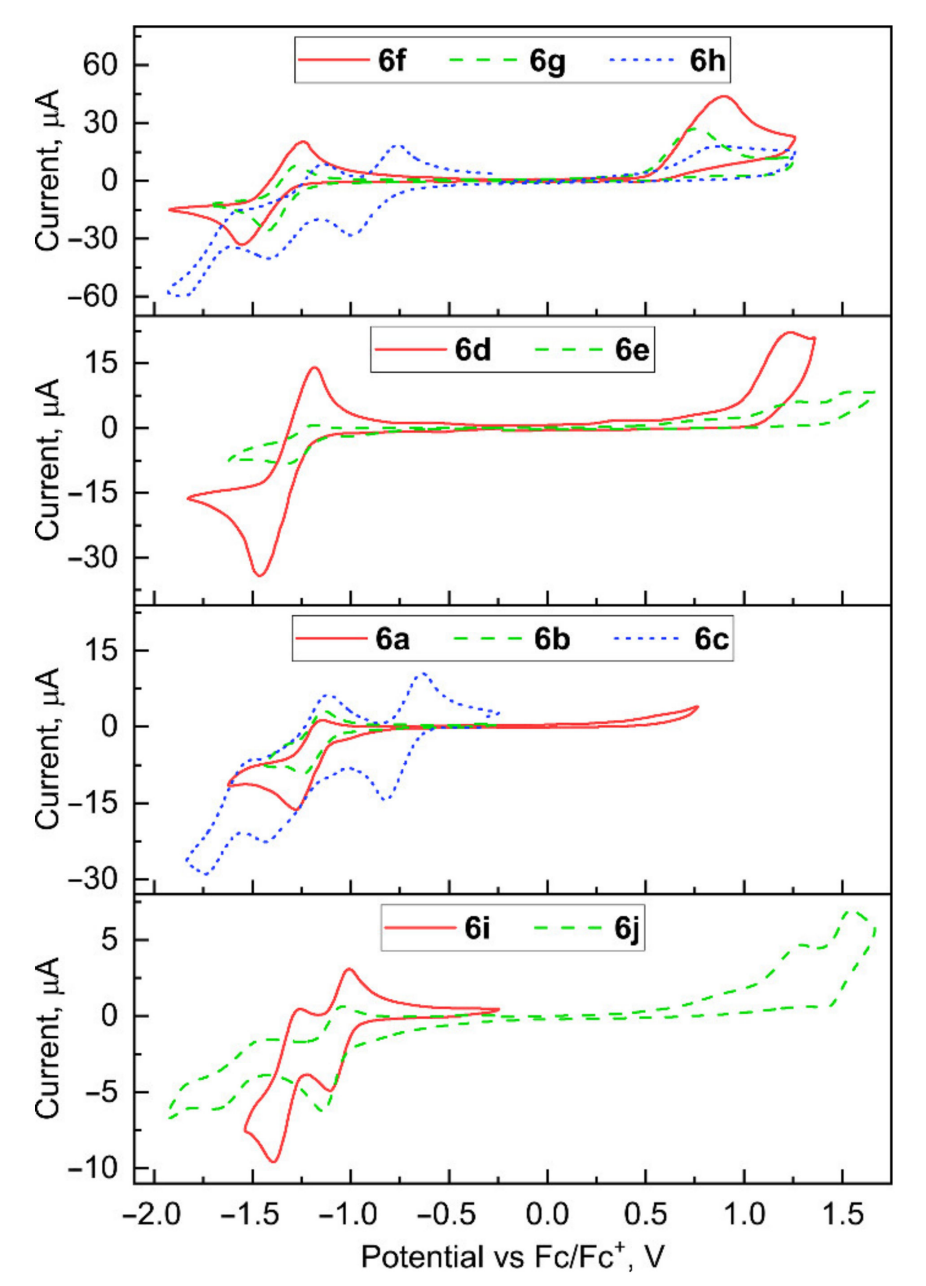
Cyclic voltammograms of BODIPYs **6a**–**j**.

The electrochemical properties of functionalized BODIPYs **6a**–**j** were recorded by cyclic voltammetry (CV) technique in dichloromethane using tetrabutylammonioumhexafluorophosphate (TBAPF_6_) as a supporting electrolyte. The representative CV plot is shown in Figure 3 and the data are summarized in Table 5.

All compounds are characterized by a reversible first reduction peak. For compounds **6c**,**h**,**i**,**j**, there are additional reduction peaks apparently related to the substituents: NO_2_ group (**6i**), CN group (**6j**), and for **6c,h** these are furazanopyrazine fragments. A number of compounds also has an irreversible oxidation peak. For NMe_2_ derivatives potential E_onset, ox_ is in the range of 0.42–0.50 V, for compound **6e** with CH_3_ substituent E_onset, ox_ is much higher and amounts to 1.02 V. The introduction of the electron-withdrawing CN group, compound **6j**, leads to an even greater increase in the oxidation potential. For **6i** containing NO_2_ group the potential could not be fixed in the accessible region.

Compounds containing furazanopyrazine substituents **6c,h** undergo reduction most easily; E_onset, red_ is −0.61 and −0.72, respectively. In general, derivatives with one acridine moiety **6a**,**d,f** are more difficult to reduce, the values of reduction potentials are in the range from −1.24 to −1.09. Compounds **6b,g** are reduced at −1.08 and −1.14 V. The presence of electron-withdrawing NO_2_ group and CN group in **6i,j** leads to a decrease in the potential to −0.97 and −0.96, respectively.

## 3. Materials and Methods

### 3.1. General

The starting materials and reagents were purchased from commercial sources and used without further purification. ^1^H-NMR (400 MHz), ^13^C-NMR (101 MHz), ^11^B-NMR (128 MHz) and ^19^F-NMR (376 MHz) spectra were recorded on an Avance II instrument (Bruker, Germany) in DMSO-d_6_ and CDCl_3_ using SiMe_4_ as internal reference. Chemical shifts (d) are reported in parts per millions (ppm) and spin multiplicities are given as singlet (s), doublet (d), triplet (t), or multiplet (m). Coupling constants (*J*) are reported in Hz. Electrospray mass spectra were recorded in positive mode with maXis impact high resolution Q-TOF mass spectrometer (Bruker Daltonics, Bremen, Germany) in 50–2500 Da mass range by direct infusion of sample solutions in methanol using kdScientific syringe pump at 120 uL/hr flow rate. The mass spectra were recorded on a GCMS-QP2010 Ultra mass spectrometer (Shimadzu, Japan) with sample ionization by electron impact (EI). The elemental analysis was carried out on an automated PE 2400 series II CHNS analyzer (Perkin Elmer, Norwalk, CT, USA).

The course of the reactions was monitored by TCL on 0.25 mm silica gel plates (60F 254). silica gel 60 (0.04–0.063 mm, Macherey-Nagel GmbH & Co, Düren, Germany) was used for column chromatography. Melting points were determined on a SMP10 melting point apparatus (Stuart, Stone, Staffordshire, United Kingdom) and are uncorrected.

Photochemical reactions were performed in a PhotoRedOx box (EvoluChem^TM^, Beverly, MA, USA) equipped with an EvoluChem™ LED, 18 W, 425 nm (HCK1012-01-012).

Dipyrromethanes **3a**–**e** were prepared according to the procedures described in the literature [44,45,46,47]. BODIPYs **7a**–**e** were prepared according to the procedures described in the literature and identified by comparing their ^1^H-NMR spectra with those given in the literature [49,50,51,52]. DDQ (0.3 mmol) in dry CH_2_Cl_2_ (2.5 mL) was added to dipyrromethane **3a**–**e** (0.3 mmol) in 20 mL of dry CH_2_Cl_2_, cooled in an ice bath under Ar atmosphere. The reaction mixture was stirred for 15 min. To the reaction mixture triethylamine (2 mL) was added immediately followed by BF_3_·Et_2_O (2 mL). The reaction mixture was further stirred for another 3 h at room temperature and washed with 0.2 M NaOH solution (50 mL) and water (100 mL). The organic layer was collected and dried over anhydrous Na_2_SO_4_, and the solvent was removed The reaction mixture was concentrated in vacuum. The residue was purified by column chromatography on silica gel.

### 3.2. Optical Measurements

All optical measurements were carried out at room temperature in luminescent quartz cells 10 mm × 10 mm. Benzene (99%, Sigma-Aldrich, Saint Louis, MO, USA), tetrachlorethylene (99%, Acros, Belgium) and ethanol (95%) were used as solvents.

The absorption spectra of the dyes were measured on a Shimadzu UV 3101PC spectrophotometer. Stationary luminescence measurements were carried out on a Shimadzu RF-6000 spectrofluorimeter, in which the manufacturer implemented the correction of obtained spectra for the spectral sensitivity of the detecting system and the spectrum of the excitation source. Light filters, which do not transmit light of higher diffraction orders on the monochromators of the instrument, were placed on the paths of the exciting and recorded beams. Nominal bandwidths of the excitation and observation monochromators were 5 nm each. Identically recorded luminescence spectra of pure solvents were subtracted from the measured luminescence spectra of the dyes. Optical densities of solutions in the luminescence measurements did not exceed 0.1 at the excitation wavelength and 0.05 in the observation band.

The luminescence quantum yields of the dyes were determined relative to an ethanol solution of rhodamine 6G, the luminescence quantum yield of which was set equal to 0.95. Excitation of the dyes was carried out near the maxima of their absorption spectra. When calculating the quantum yield of luminescence, the refractive indices of the corresponding solvents were taken into account.

### 3.3. Electrochemical Measurements

Cyclic voltammetry was carried out on an Autolab PGSTAT128N potentiostat (Metrohm, Utrecht, Netherlands) using a standard three-electrode configuration. Typically, a three electrodes cell equipped with a glass carbon working electrode, a Ag/AgNO_3_ (0.01M) reference electrode, and a glass carbon rod counter electrode was employed. The measurements were done in dichloromethane with tetrabutylammonium hexafluorophosphate (0.1 M) as the supporting electrolyte under an argon atmosphere at a scan rate of 100 mV/s. The potential of the Ag/AgNO_3_ reference electrode was calibrated by using the ferrocene/ferrocenium redox couple (Fc/Fc+).

### 3.4. General Synthesis of Dipyrromethanes **5a**–**g**

A 20-mL vial containing a solution of dipyrromethane **3a**–**e** (0.5 mmol), heterocycle **4a**,**b** (1.0 mmol), TiO_2_ (10 mass%) and acetic acid (20 mmol) in CH_2_Cl_2_ (10 mL) was treated in an ultrasonic bath for 5 min to obtain a suspension. The resulting mixture was irradiated in an Evoluchem™ PhotoRedOx box (equipped with an EvoluChem™ LED, 18 W, 425 nm) on a stirrer plate with air oxygen bubbling through the reaction mixture at room temperature (for **5a**,**c**,**d**,**f**,**h**) or 50 °C (for **5b**,**e**,**g**). The reaction was stopped after 5 h. The solvent was evaporated under reduced pressure and the crude product was purified by column chromatography on silica gel.

*1-(Acridine-9-yl)-meso-(4-bromphenyl)dipyrrometane* (**5a**). The residue was purified by column chromatography on silica gel, eluting with a mixture of hexane/ethyl acetate (8/2) to give the compound as a yellow solid (0.115 g, 48% yield), mp 216–218 °C. ^1^H-NMR (400 MHz, CDCl_3_): δ 9.75 (s, 1H), 9.23 (s, 1H), 7.59 (d, *J* = 8.7 Hz, 2H), 7.49 (d, *J* = 8.3 Hz, 2H), 7.36 (d, *J* = 8.7 Hz, 2H), 7.24 (dd, *J* = 15.7, 7.6 Hz, 4H), 7.00–6.89 (m, 2H), 6.72 (s, 1H), 6.34–6.29 (m, 1H), 6.24 (d, *J* = 2.6 Hz, 1H), 6.20–6.11 (m, 2H), 5.68 (s, 1H). ^13^C-NMR (101 MHz, CDCl_3_): δ 147.90, 141.97, 139.09, 134.94, 131.75, 131.49, 130.48, 129.98, 128.01, 126.67, 125.17, 124.61, 124.36, 121.05, 118.14, 112.60, 109.07, 108.42, 108.15, 43.64. MS (EI): *m*/*z* 479 [M+H]^+^. Anal. calcd. for C_28_H_20_BrN_3_: C, 70.30; H, 4.21; N, 8.78. Found: C, 70.40; H, 4.20; N, 8.75.

*1,9-Di(acridine-9-yl)-meso-(4-bromphenyl)dipyrrometane* (**5b**). The residue was purified by column chromatography on silica gel, eluting with a mixture of hexane/ethyl acetate (8/2) to give the compound as a red-brown solid (0.196 g, 63% yield), mp 196–198 °C. ^1^H-NMR (DMSO-*d*_6_): δ 11.62 (s, 2H), 8.16 (t, J = 9.7 Hz, 8H), 7.93–7.75 (m, 4H), 7.64 (d, *J* = 8.4 Hz, 2H), 7.59–7.50 (m, 4H), 7.42 (d, *J* = 8.4 Hz, 2H), 6.54–6.46 (m, 2H), 6.23 (t, *J* = 2.6 Hz, 2H), 5.76 (s, 1H). ^13^C-NMR (DMSO-*d*_6_): δ 148.89, 143.14, 139.53, 136.10, 131.65, 131.01, 130.54, 129.71, 127.57, 126.26, 125.43, 124.08, 120.08, 112.90, 108.64, 43.47. MS (EI): *m*/*z* 656 [M+H]^+^. Anal. calcd. for C_41_H_27_BrN_4_: C, 75.11; H, 4.15; N, 8.55. Found: C, 75.08; H, 4.17; N, 8.58.

*1,9-Di(6-phenyl-[1,2,5]oxadiazolo[3,4-b]pyrazine-5-yl)-meso-(4-bromphenyl)dipyrrometane* (**5c**). The residue was purified by column chromatography on silica gel, eluting with a mixture of hexane/ethyl acetate (9/1) to give the compound as a brown solid (0.198 g, 57% yield), mp 178–180 °C. ^1^H-NMR (CDCl_3_): δ 9.75 (s, 2H), 7.64–7.61 (m, 6H), 7.57–7.51 (m, 6H), 7.10 (d, *J* = 8.4 Hz, 2H), 5.84–5.82 (m, 2H), 5.70–5.68 (m, 2H), 5.47 (s, 1H). ^13^C-NMR (CDCl_3_): δ 163.02, 151.06, 150.70, 150.46, 139.91, 137.56, 132.44, 131.00, 129.92, 129.39, 128.71, 128.64, 122.30, 121.20, 112.24, 43.92. MS (EI): *m*/*z* 694 [M+H]^+^. Anal. calcd. for C_35_H_21_BrN_10_O_2_: C, 60.62; H, 3.05; N, 20.20. Found: 60.65; H, 3.04; N, 20.14.

*1-(Acridine-9-yl)-meso-(4-methylphenyl)dipyrrometane* (**5d**). The residue was purified by column chromatography on silica gel, eluting with a mixture of hexane/ethyl acetate (8/2) to give the compound as a brown solid (0.124 g, 60% yield), mp 230–232 °C. ^1^H-NMR (DMSO-*d*_6_): δ 11.43 (s, 1H), 10.63 (s, 1H), 8.16 (dd, *J* = 14.1, 8.7 Hz, 4H), 7.90–7.79 (m, 2H), 7.62–7.51 (m, 2H), 7.20 (q, *J* = 8.1 Hz, 4H), 6.70 (d, *J* = 1.5 Hz, 1H), 6.43 (t, *J* = 2.7 Hz, 1H), 6.09 (t, *J* = 2.7 Hz, 1H), 6.00 (d, *J* = 2.7 Hz, 1H), 5.85 (s, 1H), 5.54 (s, 1H), 2.33 (s, 3H). ^13^C-NMR (DMSO-*d*_6_): δ 148.32, 140.61, 139.33, 136.80, 135.15, 133.01, 130.01, 129.05, 128.63, 128.03, 127.18, 125.60, 124.94, 123.09, 116.92, 112.37, 107.85, 106.95, 106.15, 99.48, 43.17, 20.58. MS (EI): *m*/*z* 413 [M]^+^. Anal. calcd. for C_29_H_23_N_3_: C, 84.23; H, 5.61; N, 10.16. Found: C, 84.24; H, 5.65; N, 10.11.

*1,9-Di(acridine-9-yl)-meso-(4-methylphenyl)dipyrrometane* (**5e**). The residue was purified by column chromatography on silica gel, eluting with a mixture of hexane/ethyl acetate (8/2) to give the compound as a yellow solid (0.224 g, 76% yield), mp 235–237 °C. ^1^H-NMR (CDCl_3_): δ 9.37 (s, 2H), 7.97–7.95 (d, *J* = 8.7 Hz, 4H), 7.86–7.84 (d, *J* = 8.7 Hz, 4H), 7.49–7.45 (m, 6H), 7.33–7.31 (d, *J* = 7.8 Hz, 2H), 7.22–7.18 (m, 4H), 6.56 (s, 2H), 6.47 (s, 2H), 5.92 (s, 1H), 2.46 (s, 3H). ^13^C-NMR (DMSO-*d*_6_): δ 148.41, 139.15, 136.42, 135.41, 130.00, 129.19, 128.78, 128.15, 127.13, 125.68, 124.93, 123.31, 112.36, 107.99, 43.18, 20.65. MS (EI): *m*/*z* 591 [M+H]^+^. Anal. calcd. for C_42_H_30_N_4_: C, 85.40; H, 5.12; N, 9.48. Found: C, 85.47; H, 5.13; N, 9.51.

*1-(Acridine-9-yl)-meso-(4-N,N-dimethylaminophenyl)dipyrrometane* (**5f**). The residue was purified by column chromatography on silica gel, eluting with a mixture of hexane/ethyl acetate (8/2) to give the compound as a brown solid (0.130 g, 59% yield), mp 238–240 °C. ^1^H-NMR (DMSO-*d*_6_): δ 11.42 (s, 1H), 10.62 (s, 1H), 8.28–8.06 (m, 4H), 7.83 (t, *J* = 7.2 Hz, 2H), 7.64–7.47 (m, 2H), 7.13 (d, *J* = 8.0 Hz, 2H), 6.73 (d, *J* = 8.1 Hz, 2H), 6.67 (s, 1H), 6.41 (s, 1H), 6.06 (s, 1H), 5.98 (s, 1H), 5.82 (s, 1H), 5.43 (s, 1H), 2.88 (s, 6H). ^13^C-NMR (DMSO-*d*_6_): δ 149.12, 148.37, 139.29, 137.44, 133.63, 131.40, 129.99, 129.11, 128.61, 127.23, 125.59, 124.87, 122.86, 116.75, 112.37, 107.67, 106.84, 105.92, 42.62, 40.37. MS (EI): *m*/*z* 442 [M]^+^. Anal. calcd. for C_30_H_26_N_4_: C, 81.42; H, 5.92; N, 12.66. Found: C, 81.40; H, 5.92; N, 12.68.

*1,9-Di(acridine-9-yl)-meso-(4-N,N-dimethylaminophenyl)dipyrrometane* (**5g**). The residue was purified by column chromatography on silica gel, eluting with a mixture of hexane/benzene/ethyl acetate (1/1/1) to give the compound as a red-brown solid (0.118 g, 38% yield), mp 278–280 °C. ^1^H-NMR (DMSO-*d*_6_): δ 11.39 (s, 2H), 8.16 (d, *J* = 8.8 Hz, 8H), 7.82–7.78 (m, 4H), 7.53–7.49 (m, 4H), 7.27 (d, *J* = 8.6 Hz, 2H), 6.80 (d, *J* = 8.7 Hz, 2H), 6.47–6.45 (m, 2H), 6.23–6.21 (m, 2H), 5.64 (s, 1H), 2.91 (s, 6H). ^13^C-NMR (DMSO-*d*_6_): δ 149.84, 148.96, 139.78, 137.53, 131.53, 130.39, 129.68, 129.21, 127.67, 126.05, 125.54, 123.74, 112.89, 108.31, 43.25, 40.86. MS (EI): *m*/*z* 619 [M]^+^. Anal. calcd. for C_43_H_33_N_5_: C, 83.33; H, 5.37; N, 11.30. Found: C, 83.37; H, 5.34; N, 11.26.

*1,9-Di(6-phenyl-[1,2,5]oxadiazolo[3,4-b]pyrazine-5-yl)-meso-(4-N,N-dimethylaminophenyl)-dipyrrometane* (**5h**). The residue was purified by column chromatography on silica gel, eluting with a mixture of hexane/ethyl acetate (8/2) to give the compound as a brown solid (0.118 g, 36% yield), mp 178–180 °C. ^1^H-NMR (CDCl_3_) δ 9.78 (s, 2H), 7.65–7.61 (m, 6H), 7.57–7.53 (m, 4H), 7.08 (d, *J* = 8.7 Hz, 2H), 6.71 (d, *J* = 8.7 Hz, 2H), 5.88–5.86 (m, 2H), 5.70–5.68 (m, 2H), 5.40 (s, 1H), 2.99 (s, 6H). ^13^C-NMR (CDCl_3_): δ 163.13, 151.17, 150.70, 150.43, 150.25, 141.83, 137.74, 130.90, 128.99, 128.92, 128.71, 128.58, 125.23, 121.43, 112.89, 111.99, 43.71, 40.39. MS (EI): *m*/*z* 657 [M]^+^. Anal. calcd. for C_37_H_27_N_11_O_2_: C, 67.57; H, 4.14; N, 23.43. Found: C, 67.65; H, 4.17; N, 23.41.

*1,9-Di(acridine-9-yl)-meso-(4-nitrophenyl)dipyrrometane* (**5i**). The residue was purified by column chromatography on silica gel, eluting with a mixture of hexane/benzene/ethyl acetate (1/1/1) to give the compound as a brown solid (0.152 g, 49% yield), mp 272–274 °C. ^1^H-NMR (DMSO-*d*_6_): δ 11.70 (s, 2H), 8.33 (d, *J* = 8.7 Hz, 2H), 8.16 (dd, *J* = 15.5, 8.7 Hz, 8H), 7.86–7.82 (m, 4H), 7.73 (d, *J* = 8.7 Hz, 2H), 7.58–7.54 (m, 4H), 6.53–6.51 (m, 2H), 6.27–6.26 (m, 2H), 5.93 (s, 1H). ^13^C-NMR (DMSO-*d*_6_): δ 150.92, 148.42, 146.33, 138.90, 134.66, 130.01, 129.54, 129.24, 128.26, 126.99, 125.79, 125.00, 123.91, 123.54, 112.44, 108.38, 99.49, 43.37. MS (EI): *m*/*z* 621 [M]^+^. Anal. calcd. for C_41_H_27_N_5_O_2_: C, 79.21; H, 4.38; N, 11.27. Found: C, 79.12; H, 4.42; N, 11.31.

*1,9-Di(acridine-9-yl)-meso-(4-cyanophenyl)dipyrrometane* (**5j**). The residue was purified by column chromatography on silica gel, eluting with a mixture of hexane/ethyl acetate (8/2) to give the compound as a brown solid (0.238 g, 79% yield), mp 254–256 °C. ^1^H-NMR (CDCl_3_): δ 9.55 (s, 2H), 7.89 (d, *J* = 8.7 Hz, 4H), 7.82 (t, *J* = 8.0 Hz, 6H), 7.73 (d, *J* = 8.1 Hz, 2H), 7.51–7.41 (m, 4H), 7.23–7.14 (m, 4H), 6.57 (s, 2H), 6.44 (s, 2H), 6.02 (s, 1H). MS (EI): *m*/*z* 601 [M]^+^. Anal. calcd. for C_41_H_27_N_5_O_2_: C, 83.84; H, 4.52; N, 11.64. Found: C, 83.95; H, 4.50; N, 11.59.

### 3.5. General Procedure for BODIPYs **6a**–**g**

DDQ (0.3 mmol) in dry CH_2_Cl_2_ (2.5 mL) was added to dipyrromethane **5a**–**i** (0.3 mmol) in 20 mL of dry CH_2_Cl_2_ cooled in an ice bath under Ar atmosphere. The reaction mixture was stirred for 15 min. To the reaction mixture triethylamine (for **6a**,**d**–**i**) or diisopropylethylamine (for **6b**,**c**,**j**) (2 mL) was added immediately followed by BF_3_·Et_2_O (2 mL). The reaction mixture was further stirred for another 3 h at room temperature and washed with 0.2 M NaOH solution (50 mL) and water (100 mL). The organic layer was collected and dried over anhydrous Na_2_SO_4_, and the solvent was removed. The reaction mixture was concentrated in vacuum. The crude product was purified by column chromatography on silica gel.

*4,4-Difluoro-3-(acridine-9-yl)-8-(4-bromphenyl)-4-bora-3a,4a-diaza-s-indacene* (**6a**). The residue was purified by column chromatography on silica gel, eluting with a mixture of hexane/ethyl acetate (7/3) to give compound as a red solid (0.051 g, 32% yield), mp 218–220 °C. ^1^H-NMR (CDCl_3_): δ 8.22 (d, *J* = 8.8 Hz, 2H), 7.75–7.65 (m, 6H), 7.62 (s, 1H), 7.51 (d, *J* = 8.3 Hz, 2H), 7.46–7.37 (m, 2H), 7.09 (d, *J* = 3.9 Hz, 1H), 6.88 (d, *J* = 3.9 Hz, 1H), 6.60 (d, *J* = 3.9 Hz, 1H), 6.44 (d, *J* = 3.5 Hz, 1H). ^13^C-NMR (CDCl_3_): δ 152.15, 147.49, 144.69, 144.48, 136.10, 134.49, 133.91, 131.56, 130.96, 130.75, 129.79, 129.09, 128.64, 125.68, 125.00, 124.70, 124.48, 121.15, 118.54. ^19^F-NMR (CDCl_3_): δ-143.20. ^11^B-NMR (CDCl_3_): δ 0.34. MS (EI): *m*/*z* 525 [M+H]^+^. Anal. calcd. for C_28_H_17_BBrF_2_N_3_: C, 64.16; H, 3.27; N, 8.02. Found: C, 64.21; H, 3.20; N, 8.00.

*4,4-Difluoro-3,5-di(acridine-9-yl)-8-(4-bromphenyl)-4-bora-3a,4a-diaza-s-indacene* (**6b**). The residue was purified by column chromatography on silica gel, eluting with a mixture of hexane/ethyl acetate (8/2) to give the compound as a red solid (0.08 g, 38% yield), mp > 300 °C. ^1^H-NMR (CDCl_3_): δ 8.11 (d, *J* = 9.0 Hz, 4H), 7.87 (d, *J* = 8.1 Hz, 2H), 7.78 (d, *J* = 8.1 Hz, 2H), 7.70–7.60 (m, 8H), 7.45–7.34 (m, 4H), 7.23 (d, *J* = 4.0 Hz, 2H), 6.62 (d, *J* = 4.0 Hz, 2H). ^13^C-NMR (CDCl_3_): δ 123.31, 125.15, 125.81, 125.88, 126.56, 129.54, 129.89, 131.10, 132.10, 132.16, 132.71, 135.86, 136.68, 148.27, 154.46. ^19^F-NMR (CDCl_3_): δ-141.19. ^11^B-NMR (CDCl_3_): δ 0.65. HRMS *m*/*z* calcd. for C_41_H_25_BBrF_2_N_4_ [M+H]^+^ 701.1324, found: *m*/*z* 701.1244.

*4,4-Difluoro-3,5-di(6-phenyl-[1,2,5]oxadiazolo[3,4-b]pyrazine-5-yl)-8-(4-bromphenyl)-4-bora-3a,4a-diaza-s-indacene* (**6c**). The residue was purified by column chromatography on silica gel, eluting with a mixture of hexane/benzene/ethyl acetate (1/3/0.1) to give the compound as a dark brown solid (0.093 g, 42% yield), mp 185–187 °C. ^1^H-NMR (CDCl_3_): δ 7.76–7.73 (m, 5H), 7.54–7.49 (m, 4H), 7.44–7.39 (m, 5H), 6.89 (d, *J* = 4.4 Hz, 2H), 6.25 (d, *J* = 4.4 Hz, 2H). HRMS *m*/*z* calcd. for C_35_H_22_BBrF_2_N_11_O_2_ [M+NH]^+^ 756.1202, found: *m*/*z* 758.1182.

*4,4-Difluoro-3-(acridine-9-yl)-8-(4-methylphenyl)-4-bora-3a,4a-diaza-s-indacene* (**6d**). The residue was purified by column chromatography on silica gel, eluting with a mixture of hexane/ethyl acetate (8/2) to give the compound as a red solid (0.063 g, 46% yield), mp 246–248 °C. ^1^H-NMR (CDCl_3_): δ 8.25 (d, *J* = 8.7 Hz, 2H), 7.71 (t, *J* = 7.8 Hz, 4H), 7.60 (s, 1H), 7.54 (d, *J* = 8.0 Hz, 2H), 7.42 (dd, *J* = 8.3, 6.4 Hz, 2H), 7.33 (d, *J* = 7.8 Hz, 2H), 7.14 (d, *J* = 3.9 Hz, 1H), 6.93 (d, *J* = 4.1 Hz, 1H), 6.58 (d, *J* = 4.0 Hz, 1H), 6.42 (d, *J* = 3.7 Hz, 1H), 2.44 (s, 3H). ^13^C-NMR (CDCl_3_): δ 21.55, 119.21, 121.68, 125.67, 126.08, 126.92, 129.04, 129.37, 130.49, 130.77, 130.99, 132.17, 135.21, 135.81, 141.69, 144.89, 147.80, 151.78. ^19^F-NMR (CDCl_3_): δ-143.11. ^11^B-NMR (CDCl_3_): δ 0.37. MS (EI): *m*/*z* 459 [M]^+^. Anal. calcd. for C_29_H_20_BF_2_N_3_: C, 75.84; H, 4.39; N, 9.15. Found: C, 75.88; H, 4.40; N, 9.13.

*4,4-Difluoro-3,5-di(acridine-9-yl)-8-(4-methylphenyl)-4-bora-3a,4a-diaza-s-indacene* (**6e**). The residue was purified by column chromatography on silica gel, eluting with a mixture of hexane/ethyl acetate (8/2) to give the compound as a brown solid (0.063 g, 70% yield), mp > 300 °C. ^1^H-NMR (CDCl_3_): δ 8.00 (d, *J* = 8.7 Hz, 4H), 7.70 (d, *J* = 7.9 Hz, 2H), 7.61–7.50 (m, *J* = 16.6, 8.3 Hz, 8H), 7.41 (d, *J* = 7.8 Hz, 2H), 7.33–7.24 (m, 4H), 7.20–7.18 (m, 2H), 6.50 (d, *J* = 4.1 Hz, 2H), 2.49 (s, 3H). ^13^C-NMR (CDCl_3_): δ 21.58, 29.70, 122.87, 125.26, 125.79, 126.72, 129.46, 129.86, 130.92, 131.12, 131.36, 136.13, 137.07, 141.70, 147.50, 148.27, 153.54. ^19^F-NMR (CDCl_3_): δ-141.22. ^11^B-NMR (CDCl_3_): δ 0.69. HRMS *m*/*z* calcd. for C_35_H_22_BBrF_2_N_11_O_2_ [M+H]^+^ 637.2375, found: *m*/*z* 637.2369.

*4,4-Difluoro-3-(acridine-9-yl)-8-(4-N,N-dimethylaminophenyl)-4-bora-3a,4a-diaza-s-indacene* (**6f**). The residue was purified by column chromatography on silica gel, eluting with a mixture of hexane/ethyl acetate (8/2) to give the compound as a dark brown solid (0.086 g, 59% yield), mp 292–294 °C. ^1^H-NMR (CDCl_3_): δ 8.31 (d, *J* = 8.7 Hz, 2H), 7.85–7.76 (m, 4H), 7.72 (d, *J* = 8.8 Hz, 2H), 7.63 (s, 1H), 7.54–7.45 (m, 2H), 7.34 (d, *J* = 4.0 Hz, 1H), 7.12 (d, *J* = 4.0 Hz, 1H), 6.89 (d, *J* = 8.9 Hz, 2H), 6.68 (d, *J* = 4.0 Hz, 1H), 6.55–6.49 (m, 1H), 3.17 (s, 6H). ^13^C-NMR (CDCl_3_): δ 40.17, 111.58, 118.26, 121.08, 121.73, 125.77, 125.86, 127.11, 129.54, 130.02, 130.32, 131.24, 133.33, 134.60, 135.45, 138.15, 142.47, 148.46, 148.57, 149.97, 152.76. ^19^F-NMR (CDCl_3_): δ-143.10. ^11^B-NMR (CDCl_3_): δ 0.42. MS (EI): *m*/*z* 488 [M]^+^. Anal. calcd. for C_30_H_23_BF_2_N_4_: C, 73.79; H, 4.75; N, 11.47. Found: C, 73.83; H, 4.72; N, 11.46.

*4,4-Difluoro-3,5-di(acridine-9-yl)-8-(4-N,N-dimethylaminophenyl)-4-bora-3a,4a-diaza-s-indacene* (**6g**). The residue was purified by column chromatography on silica gel, eluting with a mixture of hexane/ethyl acetate (1/1) to give the compound as a dark brown solid (0.112 g, 56% yield), mp > 300 °C. ^1^H-NMR (CDCl_3_): δ 8.11 (d, *J* = 8.7 Hz, 4H), 7.84 (d, *J* = 8.7 Hz, 2H), 7.71 (d, *J* = 8.6 Hz, 4H), 7.68–7.60 (m, 4H), 7.42–7.33 (m, 6H), 6.93 (d, *J* = 8.8 Hz, 2H), 6.59 (d, *J* = 4.0 Hz, 2H), 3.16 (s, 6H). ^13^C-NMR (CDCl_3_): δ 40.15, 111.63, 121.76, 122.12, 125.51, 125.60, 126.99, 129.37, 129.80, 130.67, 133.48, 135.71, 137.78, 148.31, 151.13, 152.78. ^19^F-NMR (CDCl_3_): δ-140.95. ^11^B-NMR (CDCl_3_): δ 0.75. HRMS *m*/*z* calcd. for C_43_H_31_BBrF_2_N_5_ [M+H]^+^ 666.2641, found: *m*/*z* 665.2560.

*4,4-Difluoro-3,5-di(6-phenyl-[1,2,5]oxadiazolo[3,4-b]pyrazine-5-yl)-8-(4-N,N-dimethylamino-phenyl)-4-bora-3a,4a-diaza-s-indacene* (**6h**). The residue was purified by column chromatography on silica gel, eluting with a mixture of hexane/ethyl acetate (1/1) to give the compound as a dark brown solid (0.11 g, 52% yield), mp 206–208 °C. ^1^H-NMR (CDCl_3_): δ 7.76–7.68 (m, 4H), 7.62 (d, *J* = 8.9 Hz, 2H), 7.47 (t, *J* = 7.5 Hz, 2H), 7.37 (t, *J* = 7.6 Hz, 4H), 6.97 (d, *J* = 4.3 Hz, 2H), 6.83 (d, *J* = 9.0 Hz, 2H), 6.22 (d, *J* = 4.3 Hz, 2H), 3.16 (s, 6H). ^13^C-NMR (CDCl_3_): δ 40.17, 111.90, 121.82, 122.11, 128.47, 129.92, 130.41, 131.26, 134.77, 136.47, 136.64, 149.95, 150.55, 150.69, 151.18, 153.83, 155.56, 162.65. ^19^F-NMR (CDCl_3_): δ-130.60. ^11^B-NMR (CDCl_3_): δ 1.19. MS (EI): *m*/*z* 703 [M]^+^. Anal. calcd. for C_37_H_22_BBrF_2_N_11_O_2_: C, 63.17; H, 3.44; N, 21.90. Found: C, 63.20; H, 3.43; N, 21.88.

*4,4-Difluoro-3,5-di(acridine-9-yl)-8-(4-nitrophenyl)-4-bora-3a,4a-diaza-s-indacene* (**6i**). The residue was purified by column chromatography on silica gel, eluting with a mixture of hexane/benzene/ethyl acetate (2/2/1) to give the compound as a red solid (0.096 g, 48% yield), mp >300 °C. ^1^H-NMR (DMSO-*d*_6_): δ 8.60 (d, *J* = 8.1 Hz, 2H), 8.31 (d, *J* = 8.5 Hz, 2H), 8.06 (d, *J* = 8.6 Hz, 4H), 7.81–7.68 (m, 4H), 7.60–7.45 (m, 8H), 7.41 (d, *J* = 3.9 Hz, 2H), 6.92 (d, *J* = 3.7 Hz, 2H). ^13^C-NMR (DMSO-*d*_6_): δ 123.80; 124.40; 126.27; 126.54; 127.95; 128.99; 130.32; 136.53; 147.58; 153.59; 155.71. MS (EI): *m*/*z* 667 [M]^+^. Anal. calcd. for C_41_H_24_BF_2_N_5_O_2_: C, 73.78; H, 3.62; N, 10.49. Found: C, 73.80; H, 3.60; N, 10.48.

*4,4-Difluoro-3,5-di(acridine-9-yl)-8-(4-cyanophenyl)-4-bora-3a,4a-diaza-s-indacene* (**6j**). The residue was purified by column chromatography on silica gel, eluting with a mixture of hexane/ethyl acetate (1/1) to give the compound as a red-brown solid (0.089 g, 46% yield), mp 229–231 °C. ^1^H-NMR (CDCl_3_): δ 8.12 (d, *J* = 8.7 Hz, 4H), 8.02 (s, 4H), 7.72–7.60 (m, *J* = 13.0, 7.9 Hz, 8H), 7.45–7.36 (m, 4H), 7.16 (d, *J* = 4.1 Hz, 2H), 6.64 (d, *J* = 4.1 Hz, 2H). HRMS *m*/*z* calcd. for C_43_H_31_BBrF_2_N_5_ [M+H]^+^ 648.2171, found: *m*/*z* 648.2178.

## 4. Conclusions

An approach for the oxidative C-H/C-H coupling of dipyrromethanes with azines in the presence of the heterogeneous O_2_/TiO_2_/visible light irradiation photocatalytic system was proposed for the first time. Mono- and disubstituted dipyrromethanes were used in the synthesis of new derivatives of BODIPY family fluorophores. Fifteen derivatives of 4,4-difluoro-8-phenyl-4-boron-3*a*,4*a*-diaza-*s*-indacene were synthesized, 10 of which are new compounds. The introduction of azinyl substituents at the positions 3 and 5 of the meso-aryl substituted BODIPYs leads in most cases to a red shift of the absorption and emission spectra and to an increase in the photoluminescence quantum yield. Dye containing furazanopyrazine groups at the positions 3 and 5 of the meso-bromophenyl substituted BODIPY exhibits an increased fluorescence quantum yield (0.81 in benzene) compared with BODIPY without an azine substituent (0.036 in benzene).

## Figures and Tables

**Table 1:** Optimization of reaction conditions ^1^.

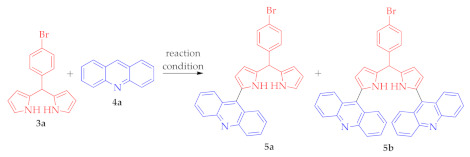					
Entry	Acid	Solution	Temperature, °C	5a [%] ^2^	5b [%] ^2^
1	CH_3_COOH	-	rt	23	0
2	CH_3_COOH	-	120	0	0
3	CF_3_COOH	CH_2_Cl_2_	rt	0	0
4	BF_3_OEt_2_	CH_2_Cl_2_	rt	0	0
5	CH_3_COOH	CH_2_Cl_2_	rt	48	0
6	CH_3_COOH	CH_2_Cl_2_	30	40	10
7	CH_3_COOH	CH_2_Cl_2_	45	10	40
8	CH_3_COOH	CH_2_Cl_2_	50	0	63
9	CH_3_COOH	CH_2_Cl_2_	>50	0	0

^1^ General conditions: **3a** (0.5 mmol), **4a** (1 mmol), TiO_2_ catalyst (10 mass%), an appropriate acid and solvent were irradiated with a 425 nm blue LED (18 W; 33 mW/cm^2^) with air oxygen bubbling through the reaction mixture. ^2^ Isolated yields.

**Table 2:** Synthetic procedure and yields of dipyrromethane **5a**–**o**.

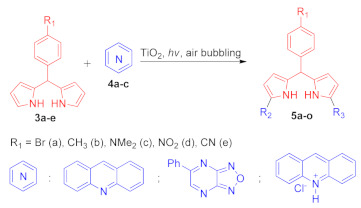							
Compound	R_1_	R_2_	R_3_	Additive	Solution	Temperature, °C	Yield, %
**5a**	Br	acridine	H	CH_3_COOH	CH_2_Cl_2_	rt	48 ^1^
**5b**	Br	acridine	acridine	CH_3_COOH	CH_2_Cl_2_	50	63 ^1^
**5c**	Br	6-phenyl-[1,2,5]oxadiazolo[3,4-*b*]pyrazine	6-phenyl-[1,2,5]oxadiazolo[3,4-*b*]pyrazine	CH_3_COOH	CH_2_Cl_2_	rt	57 ^1^
**5d**	CH_3_	acridine	H	CH_3_COOH	CH_2_Cl_2_	rt	60 ^1^
**5e**	CH_3_	acridine	acridine	CH_3_COOH	CH_2_Cl_2_	50	76 ^1^
**5f**	NMe_2_	acridine	H	CH_3_COOH	CH_2_Cl_2_	rt	59 ^1^
**5g**	NMe_2_	acridine	acridine	CH_3_COOH	CH_2_Cl_2_	50	38 ^1^
**5h**	NMe_2_	6-phenyl-[1,2,5]oxadiazolo[3,4-*b*]pyrazine	6-phenyl-[1,2,5]oxadiazolo[3,4-*b*]pyrazine	CH_3_COOH	CH_2_Cl_2_	rt	36 ^1^
**5i**	NO_2_	acridine	acridine	CH_3_COOH	CH_2_Cl_2_	rt	49 ^1^
**5j**	CN	acridine	acridine	CH_3_COOH	CH_2_Cl_2_	rt	79 ^1^
**5k**	Br	acridine hydrochloride	acridine hydrochloride	-	*n*-BuOH	rt	85 ^2^
**5l**	CH_3_	acridine hydrochloride	acridine hydrochloride	-	*n*-BuOH	rt	80 ^2^
**5m**	NMe_2_	acridine hydrochloride	acridine hydrochloride	-	*n*-BuOH	rt	82 ^2^
**5n**	NO_2_	acridine hydrochloride	acridine hydrochloride	-	*n*-BuOH	rt	85 ^2^
**5o**	CN	acridine hydrochloride	acridine hydrochloride	-	*n*-BuOH	rt	85 ^2^

^1^ Isolated yields. ^2^ The yield was determined by NMR spectroscopy.

**Table 3:** Synthesis of BODIPY **6a**–**j**.

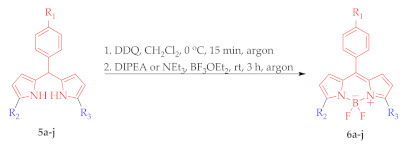					
Compound	R_1_	R_2_	R_3_	Base	Yield, % ^1^
**6a**	Br	acridine	H	NEt_3_	32
**6b**	Br	acridine	acridine	DIPEA	38
**6c**	Br	6-phenyl-[1,2,5]oxadiazolo[3,4-*b*]pyrazine	6-phenyl-[1,2,5]oxadiazolo[3,4-*b*]pyrazine	DIPEA	42
**6d**	CH_3_	acridine	H	NEt_3_	46
**6e**	CH_3_	acridine	acridine	NEt_3_	70
**6f**	NMe_2_	acridine		NEt_3_	59
**6g**	NMe_2_	acridine	acridine	NEt_3_	56
**6h**	NMe_2_	6-phenyl-[1,2,5]oxadiazolo[3,4-*b*]pyrazine	6-phenyl-[1,2,5]oxadiazolo[3,4-*b*]pyrazine	NEt_3_	52
**6i**	NO_2_	acridine	acridine	NEt_3_	48
**6j**	CN	acridine	acridine	DIPEA	46

^1^ Isolated yields.

**Table 4:** The photophysical properties of the synthesized dyes in tetrachlorethylene and benzene: the position of the long-wavelength peak of the absorption spectrum λ_abs_, the position of the peak of the luminescence spectrum λ_em_, the quantum yield of luminescence φ.

Compound	Solvent	λ_abs_/nm	λ_em_/nm	*φ*
**7a**	C_2_Cl_4_	500	536	0.284
**7a**	benzene	499	635	0.049
**6f**	C_2_Cl_4_	510	570	0.319
**6f**	benzene	508	637	0.054
**6g**	C_2_Cl_4_	520	600	0.289
**6g**	benzene	519	634	0.068
**6h**	benzene	557	782	0.0025
**7b**	C_2_Cl_4_	503	519	0.063
**7b**	benzene	503	519	0.063
**6d**	C_2_Cl_4_	513	576	0.155
**6d**	benzene	512	581	0.140
**6e**	C_2_Cl_4_	524	611	0.249
**6e**	benzene	523	614	0.228
**7c**	C_2_Cl_4_	507	525	0.027
**7c**	benzene	506	525	0.036
**6a**	C_2_Cl_4_	517	589	0.077
**6a**	benzene	516	594	0.082
**6b**	C_2_Cl_4_	529	626	0.160
**6b**	benzene	527	628	0.160
**6c**	C_2_Cl_4_	556	613	0.70
**6c**	benzene	555	619	0.81
**7d**	C_2_Cl_4_	511	535	0.0072
**7d**	benzene	510	537	0.0078
**6j**	C_2_Cl_4_	533	640	0.055
**6j**	benzene	512	544	0.061
**7e**	C_2_Cl_4_	513	541	0.0058
**7e**	benzene	512	544	0.0064
**6i**	C_2_Cl_4_	535	646	0.036
**6i**	benzene	533	649	0.041

**Table 5:** Electrochemical data for compounds **6a**–**j** in CH_2_Cl_2_.

Compound	E_[onset, red vs. Fc/Fc_^+^_],_ V	E_LUMO_^1^_,_ eV	E_[onset, ox vs. Fc/Fc_^+^_],_ V	E_HOMO_^2^_,_ eV	E_gap,_ eV
**6a**	−1.09	−4.01	-		
**6b**	−1.08	−4.02	-		
**6c**	−0.61	−4.49	-		
**6d**	−1.16	−3.94	0.99	−6.09	2.15
**6e**	−1.13	−3.97	1.02	6.12	2.15
**6f**	−1.24	−3.86	0.50	−5.60	1.74
**6g**	−1.14	−3.96	0.42	−5.52	1.56
**6h**	−0.72	−4.38	0.48	−5.58	1.2
**6i**	−0.97	−4.13	-		
**6j**	−0.96	−4.14	1.07	6.17	2.03

^1^ E_LUMO_ = −(E_[onset, red vs. Fc/Fc_^+^_]_ + 5.1) (eV). ^2^ E_HOMO_ = −(E_[onset, ox vs. Fc/Fc_^+^_]_ + 5.1) (eV).

## Data Availability

The data presented in this study are available in the article and Supplementary Materials.

## Supplementary Materials

The following are available online: NMR spectra of compounds **5a**–**j** and **6a**–**j**.

## Sample Availability

Samples of the compounds **6**–**j** are available from the authors.
