# Supplementary material for: Oxidative C-H/C-H Coupling of Dipyrromethanes with Azines by TiO2-Based Photocatalytic System. Synthesis of New BODIPY Dyes and Their Photophysical and Electrochemical Properties
Source: Molecules. 2021 Sep 13;26(18):5549. doi: 10.3390/molecules26185549 (PMC8466589; doi:10.3390/molecules26185549)
Supplement: Supplementary file 1 [file molecules-26-05549-s001.zip › molecules-1362141-supplementary.pdf]

# Oxidative C-H/C-H Coupling of Dipyrromethanes with Azines by $TiO_2$-Based Photocatalytic System. Synthesis of New BODIPY Dyes and Their Photophysical and Electrochemical Properties

**Maria A. Trestsova [1], Irina A. Utepova [1,2,*], Oleg N. Chupakhin [1,2], Maksim V. Semenov [1], Dmitry N. Pevtsov [3,4], Lyubov M. Nikolenko [4], Sergey A. Tovstun [3,4], Anna V. Gadomska [4], Alexander V. Shchepochkin [2], Gregory A. Kim [2], Vladimir F. Razumov [3,4], Irina B. Dorosheva [1,5] and Andrey A. Rempel [1,5]**

1 Department of Organic and Biomolecular Chemistry, Ural Federal University, 19 Mira Street, 620002 Ekaterinburg, Russia; maria.trestcova@urfu.ru (M.A.T.); chupakhin@ios.uran.ru (O.N.C.); semmaximwork@gmail.com (M.V.S.); i.b.dorosheva@urfu.ru (I.B.D.); rempel.imet@mail.ru (A.A.R.)
2 Institute of Organic Synthesis of the Russian Academy of Sciences, 22 S. Kovalevskoy Street, 620108 Ekaterinburg, Russia; sasha-mmm@mail.ru (A.V.S.); kim-g@ios.uran.ru (G.A.K.)
3 Moscow Institute of Physics and Technology, 9 Institutsky Lane, 141701 Dolgoprudny, Russia; pevtsovdm@gmail.com (D.N.P.); tovstun@icp.ac.ru (S.A.T.); razumov@icp.ac.ru (V.F.R.)
4 Institute of Problems of Chemical Physics of the Russian Academy of Sciences, 1 Academician Semenov Avenue, 142432 Chernogolovka, Russia; nav@icp.ac.ru (L.M.N.); ann.gadomsky@gmail.com (A.V.G.)
5 Institute of Metallurgy of the Ural Branch of the Russian Academy of Sciences, 101 Amundsena Street, 620016 Ekaterinburg, Russia
* Correspondence: i.a.utepova@urfu.ru

# Supplementary Information

## Table of Contents

NMR spectra of compounds **5a-j** S2

NMR spectra of compounds **6a-j** S21

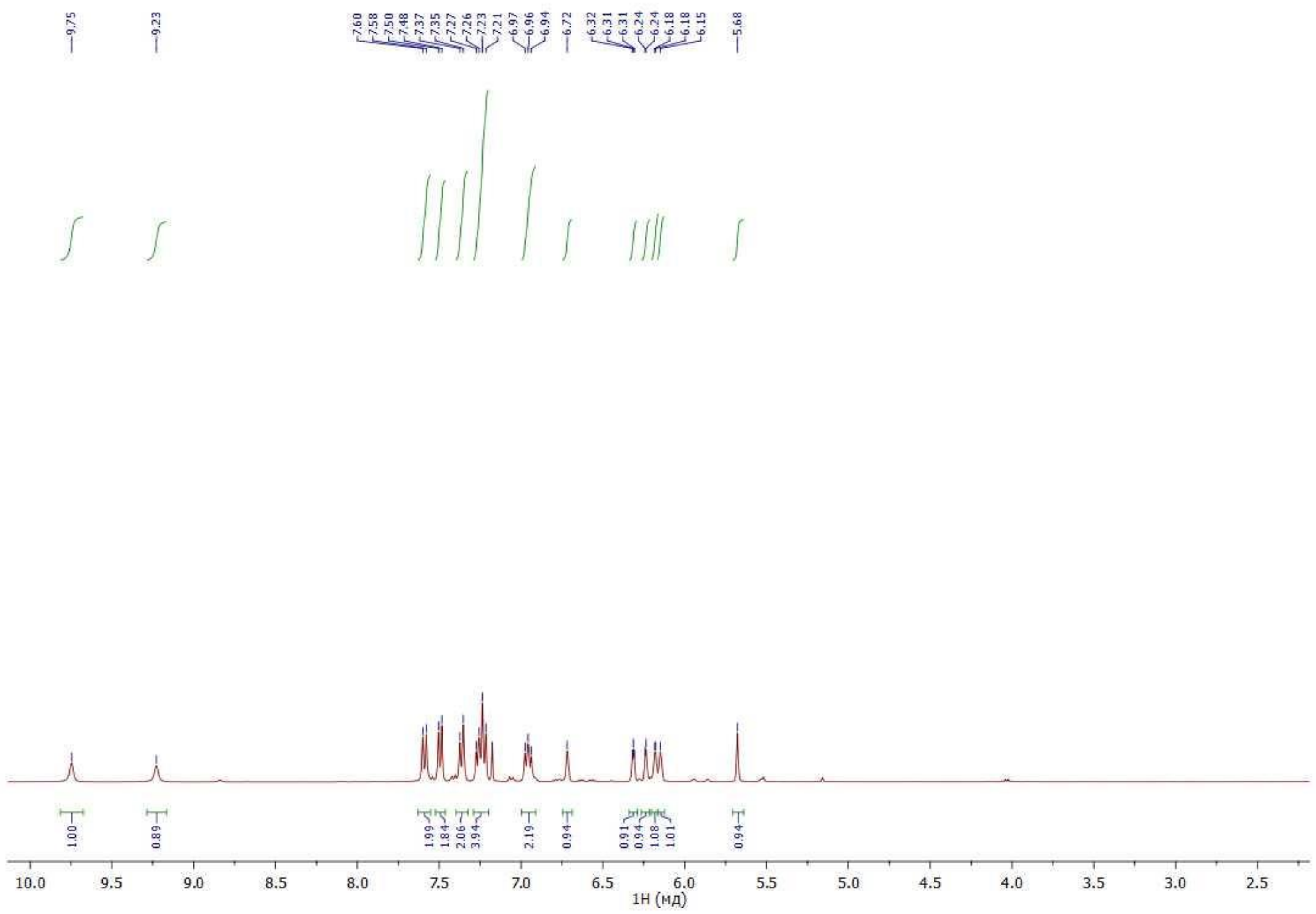


**Figure S1. $^{1}$H NMR Spectrum of 5a**

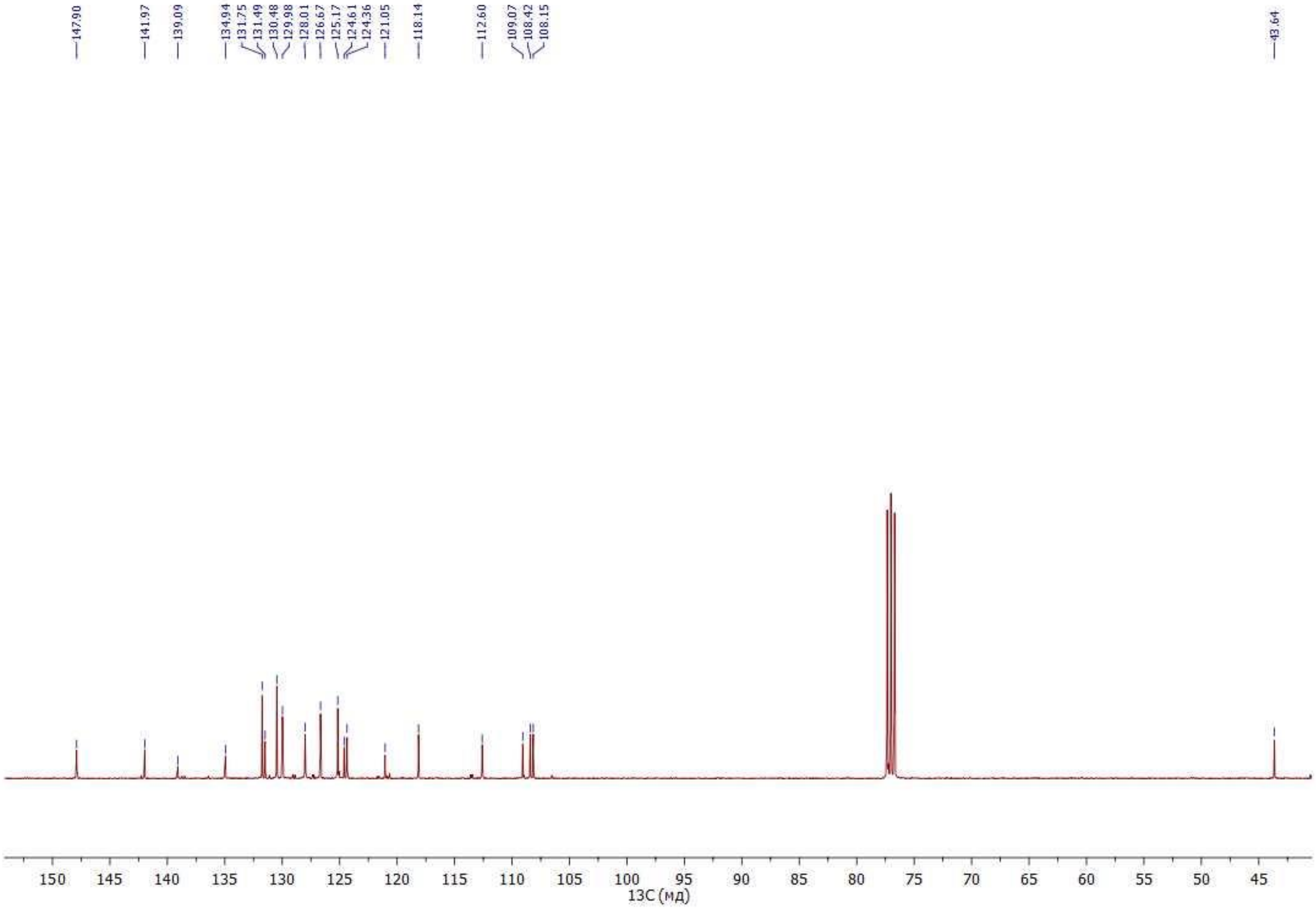


**Figure S2.** **$^{13}$C NMR Spectrum of 5a**

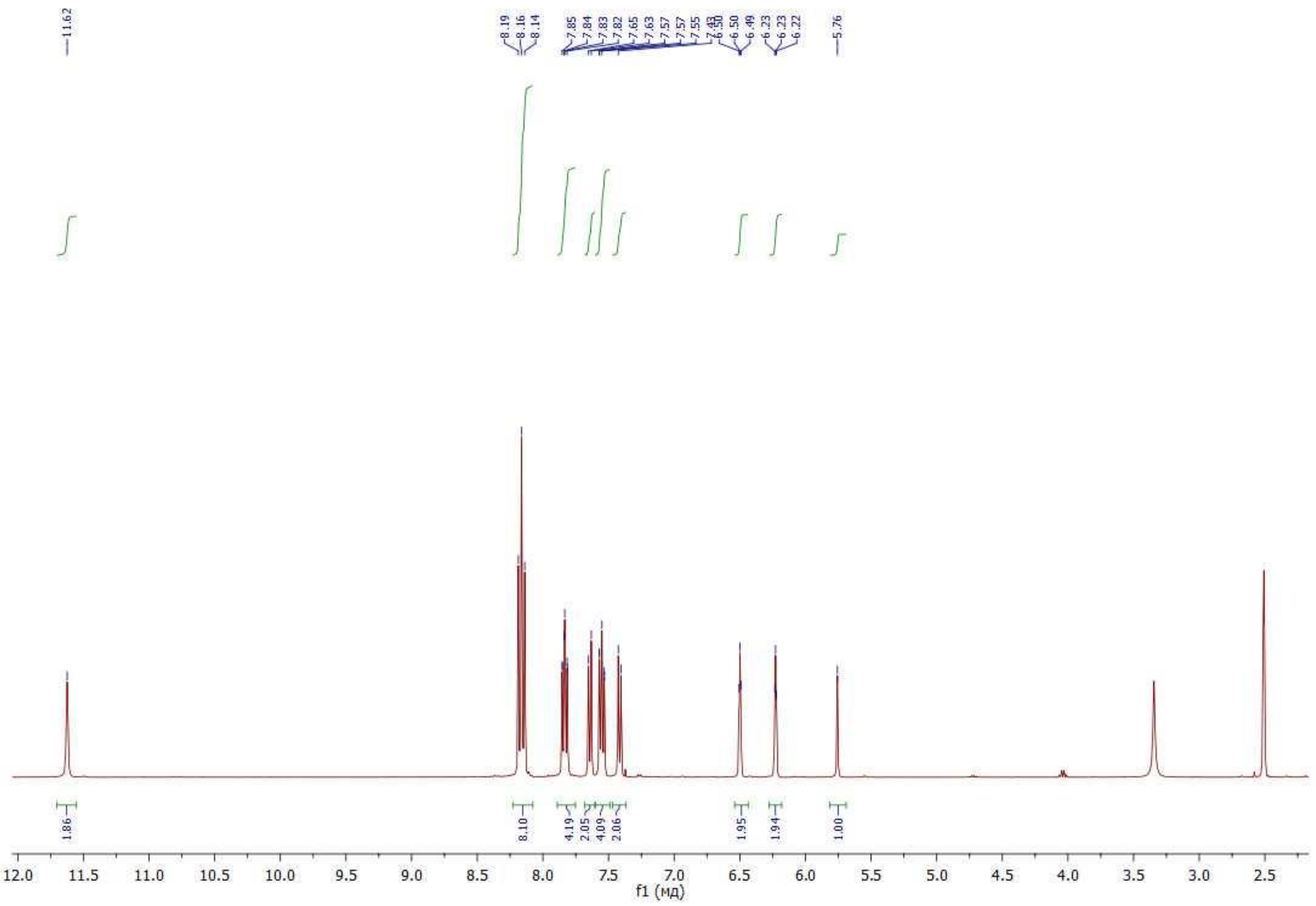

**Figure S3.** $^{1}$**H NMR Spectrum of 5b**

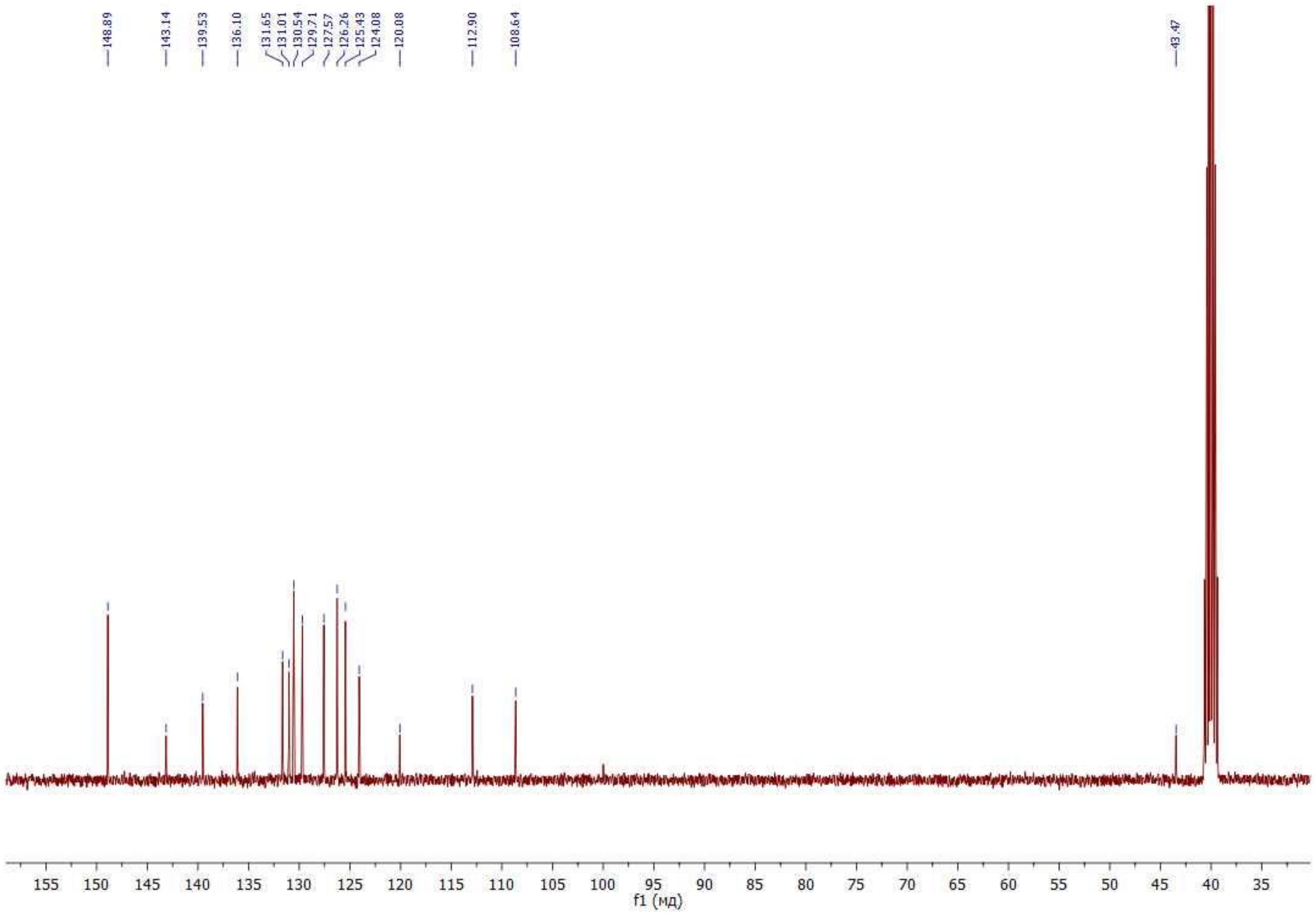


**Figure S4. $^{13}$C NMR Spectrum of 5b**

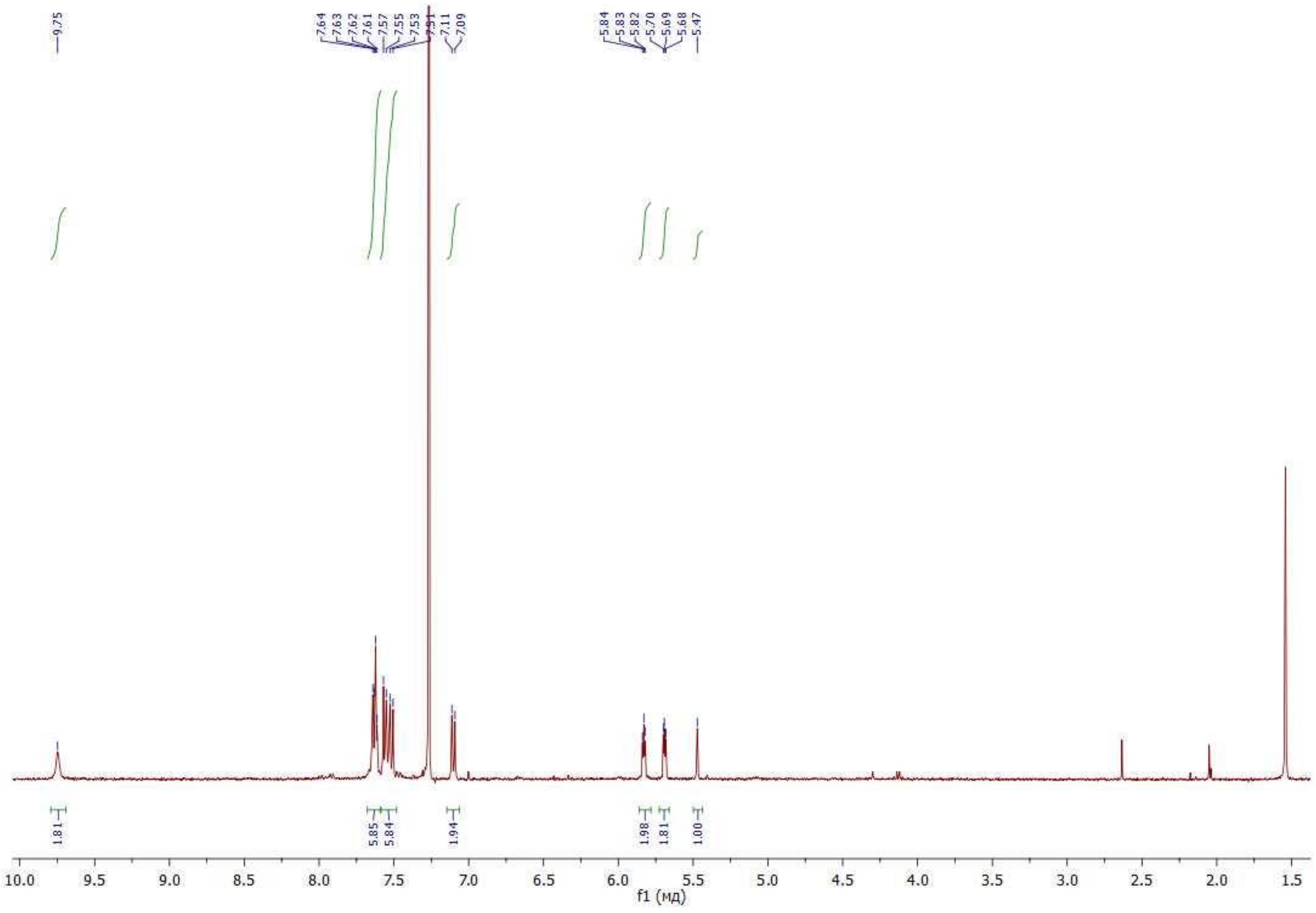


**Figure S5. $^{1}$H NMR Spectrum of 5c**

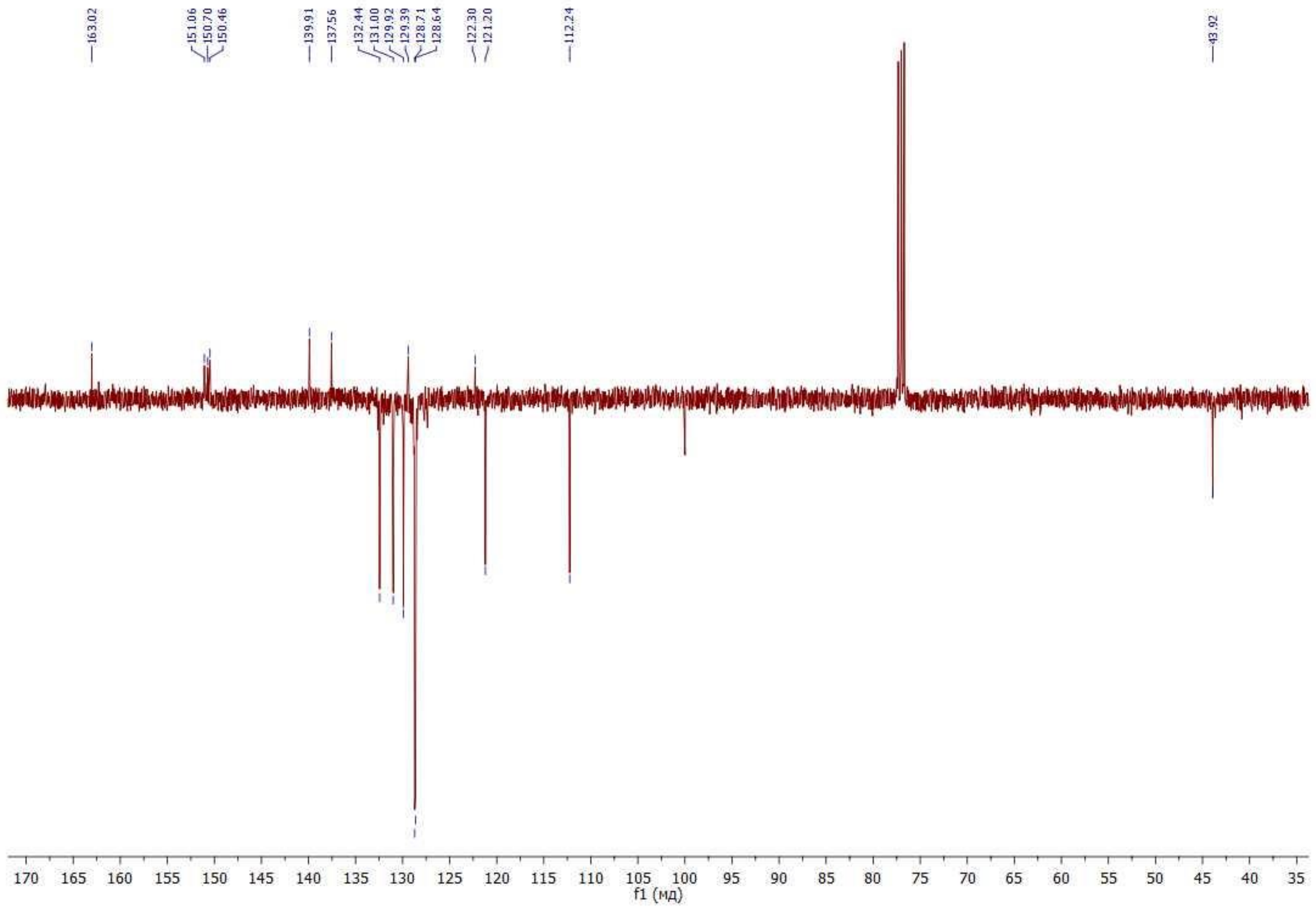


**Figure S6. $^{13}C$ NMR Spectrum of 5c**

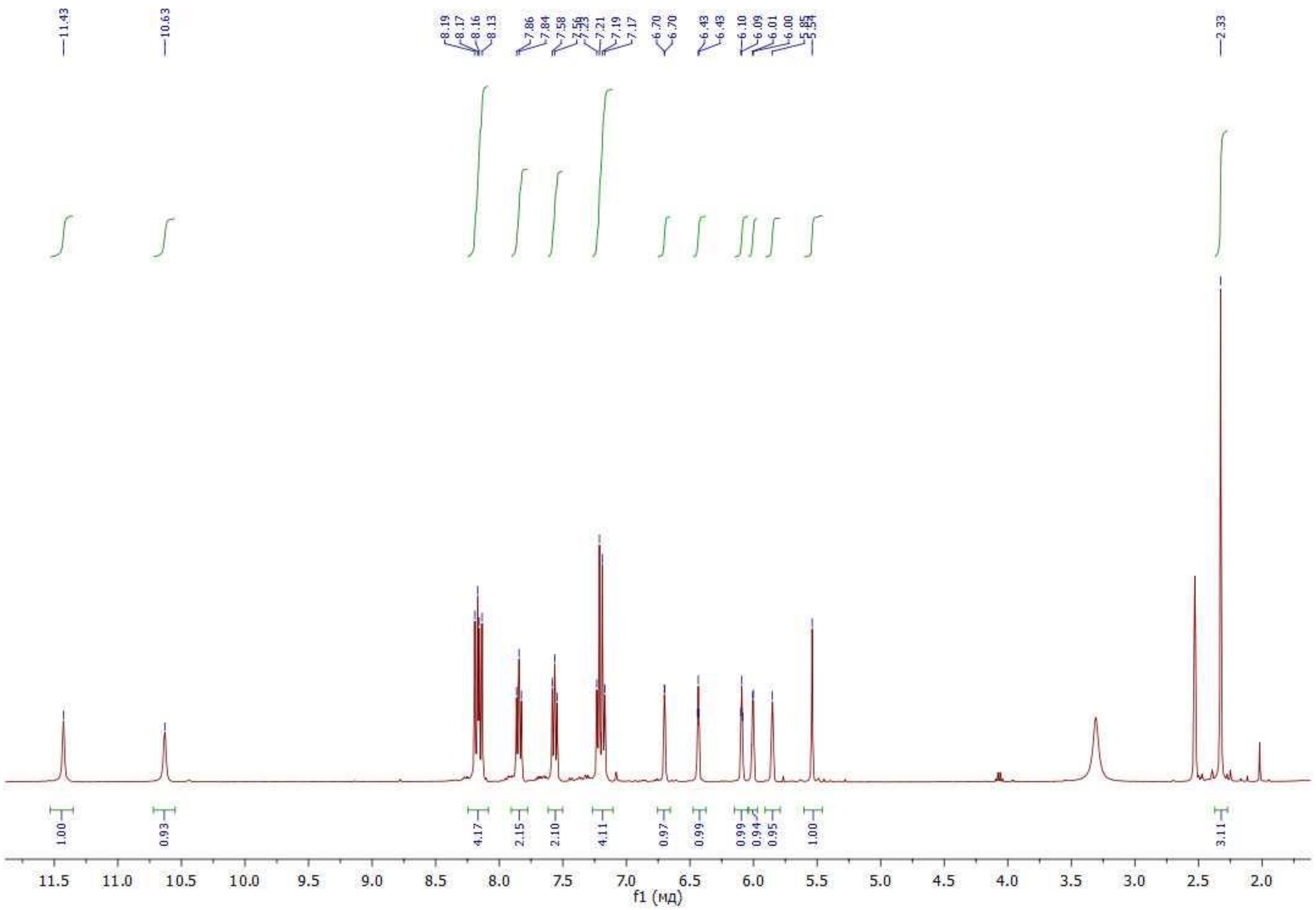

**Figure S7. ¹H NMR Spectrum of 5d**

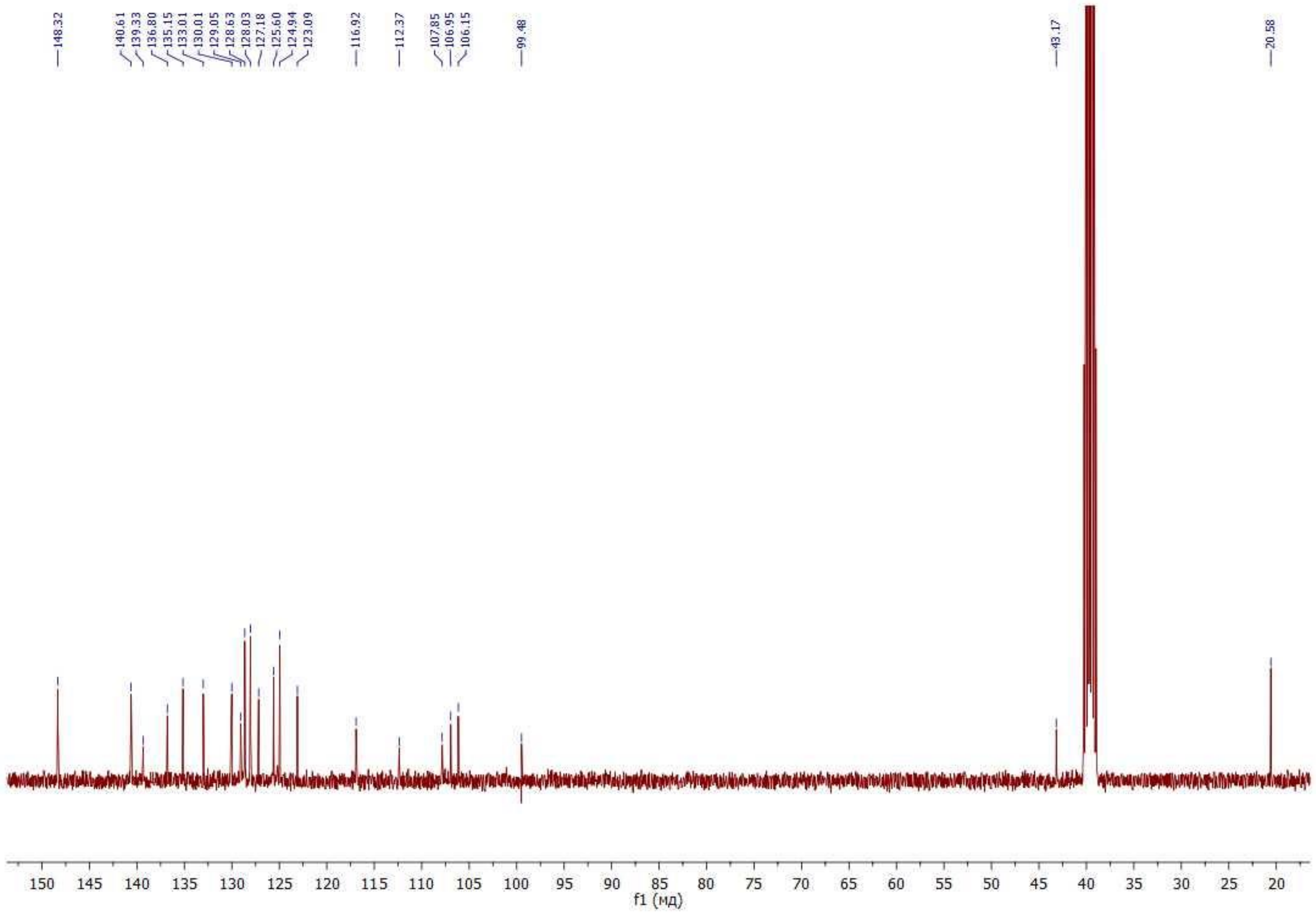


**Figure S8. ^13^C NMR Spectrum of 5d**

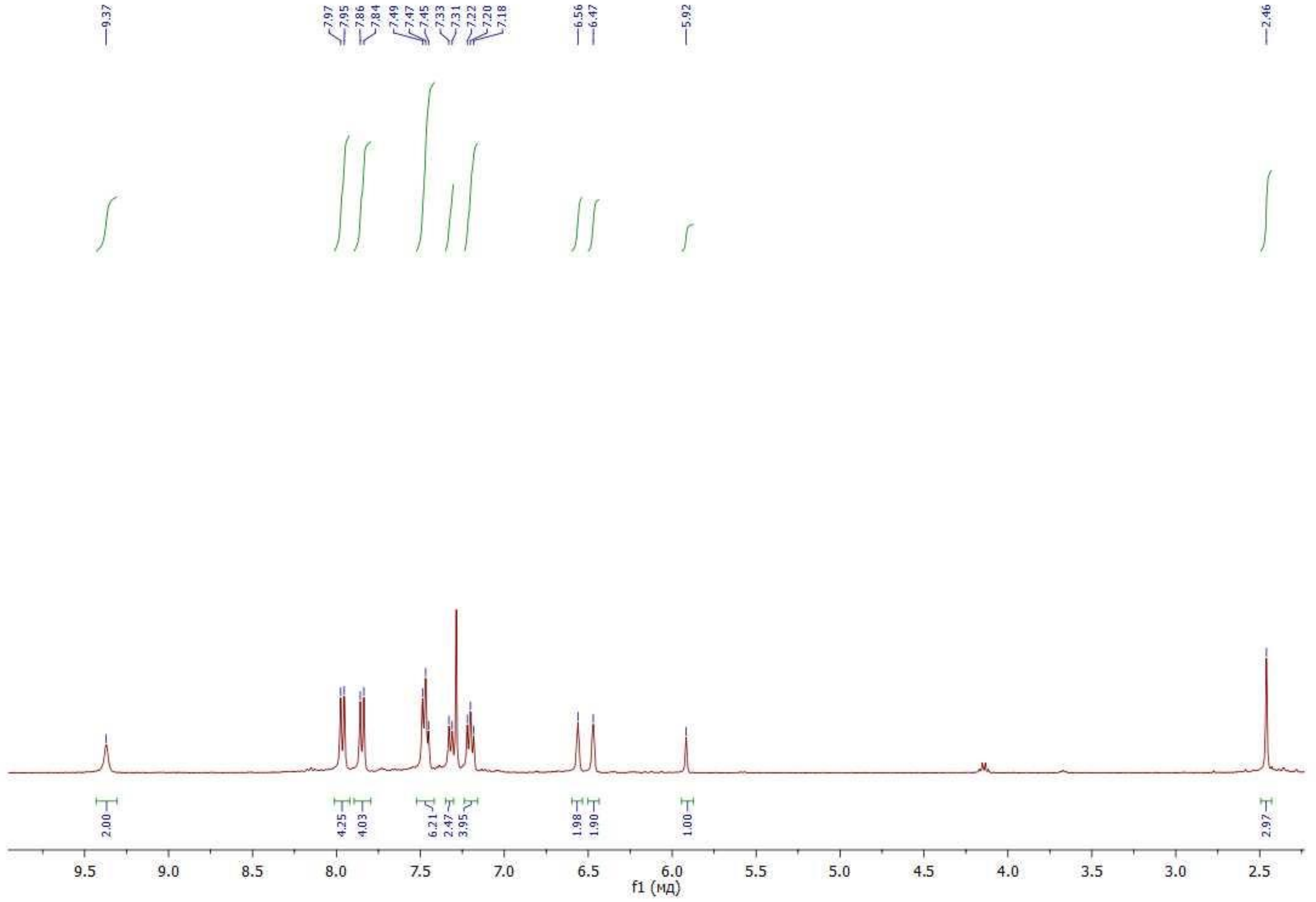


**Figure S9. ¹H NMR Spectrum of 5e**

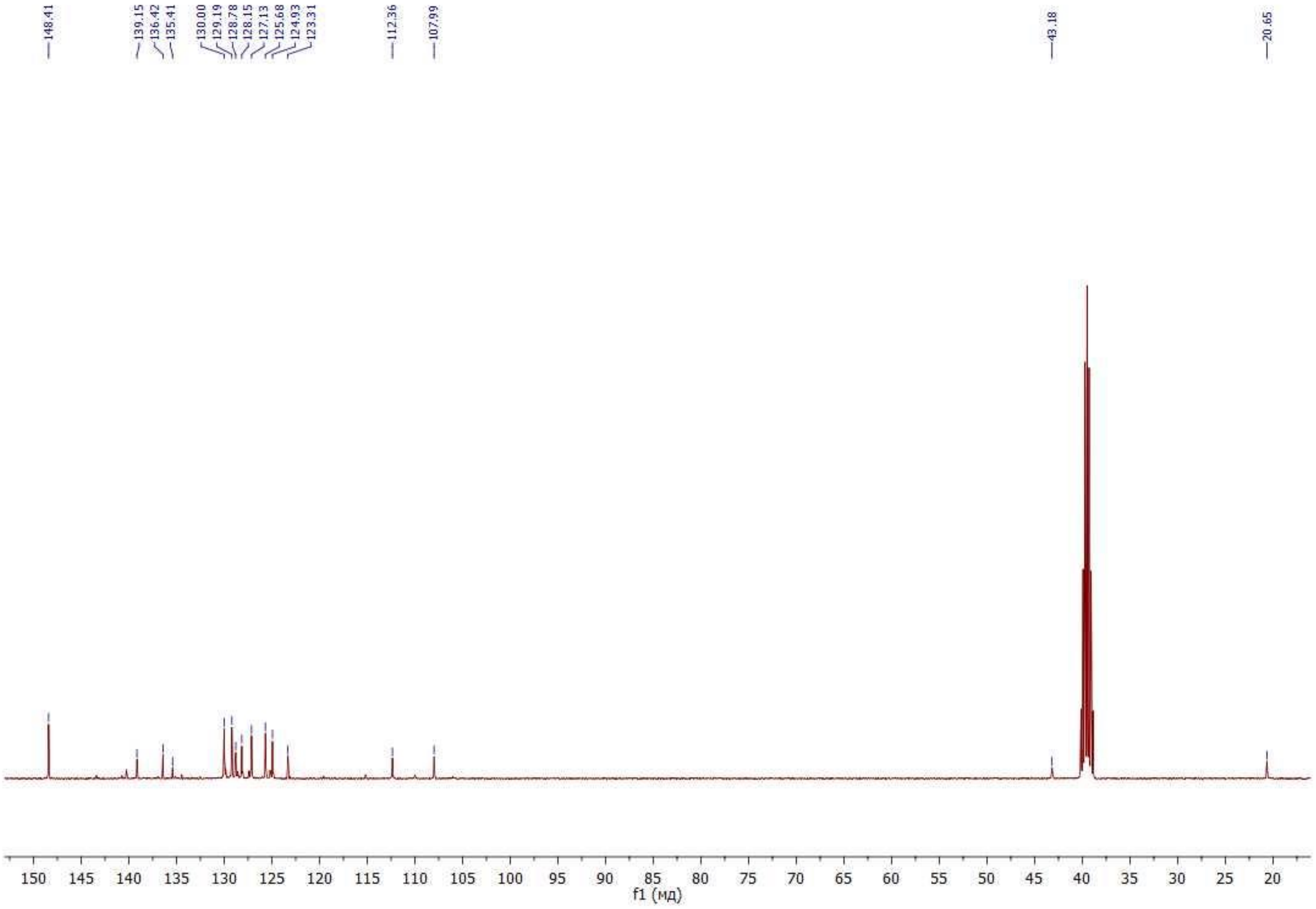


**Figure S10. $^{13}$C NMR Spectrum of 5e**

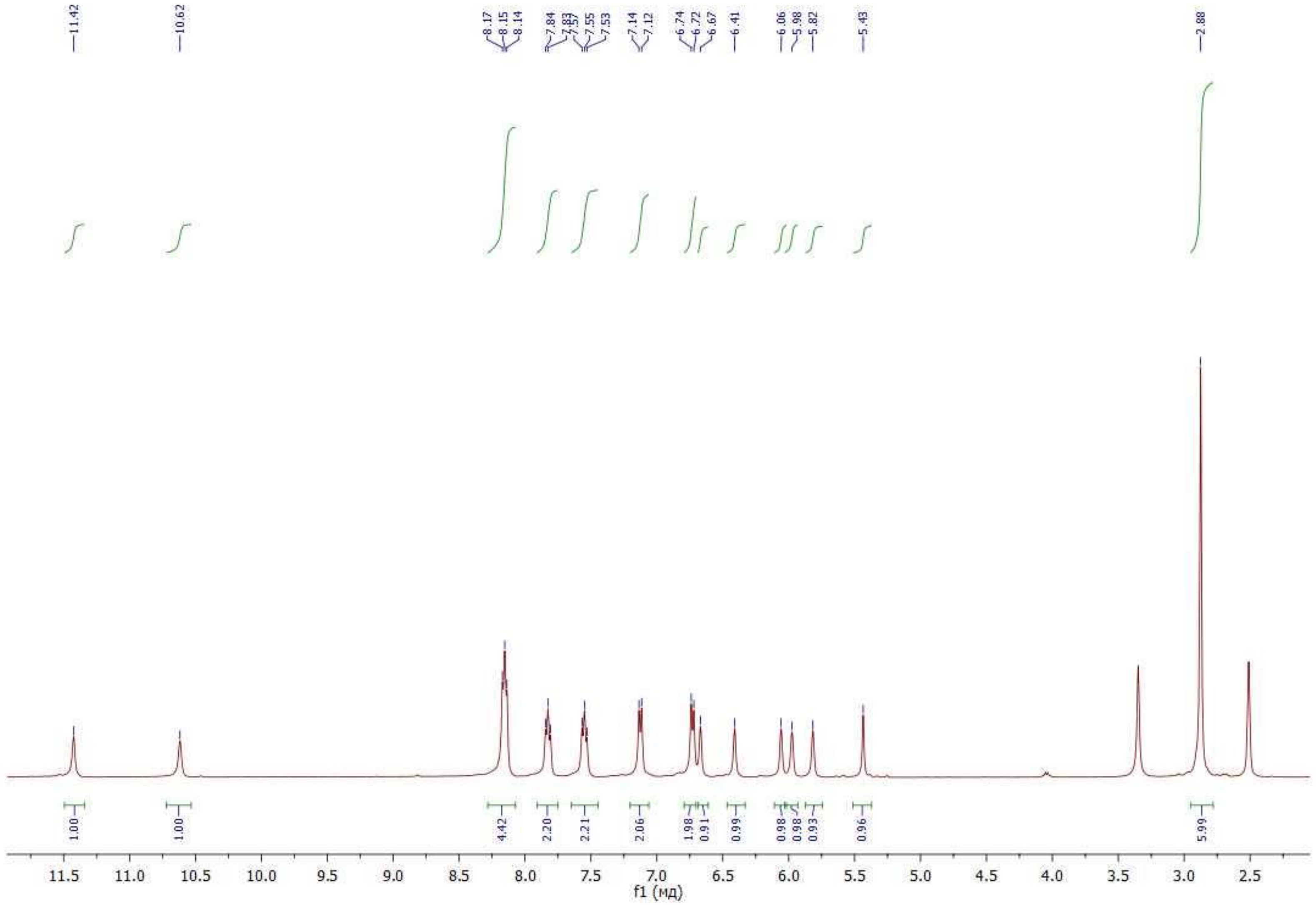


**Figure S11.** ¹H NMR Spectrum of 5f

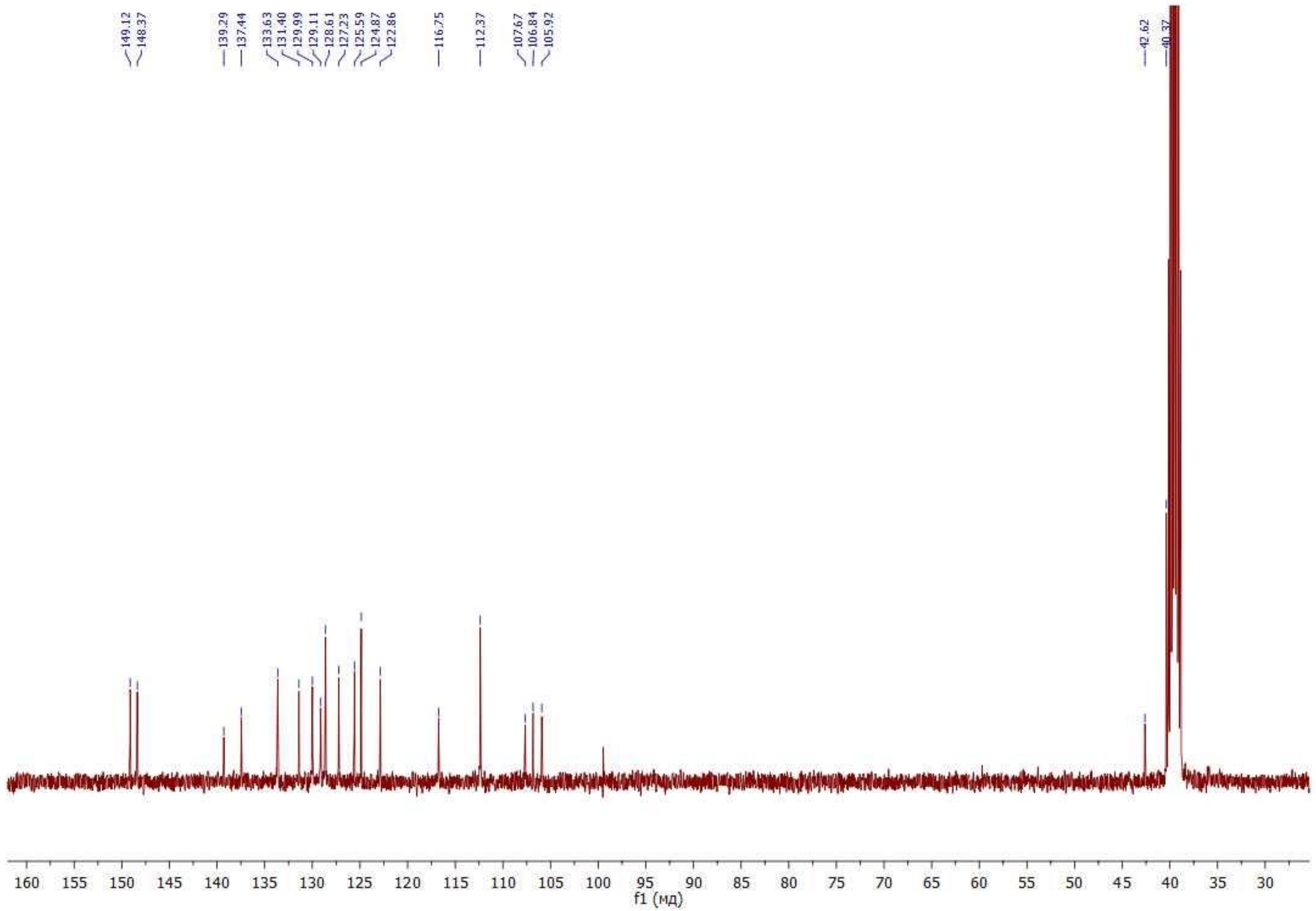


**Figure S12.** $^{13}$C **NMR Spectrum of 5f**

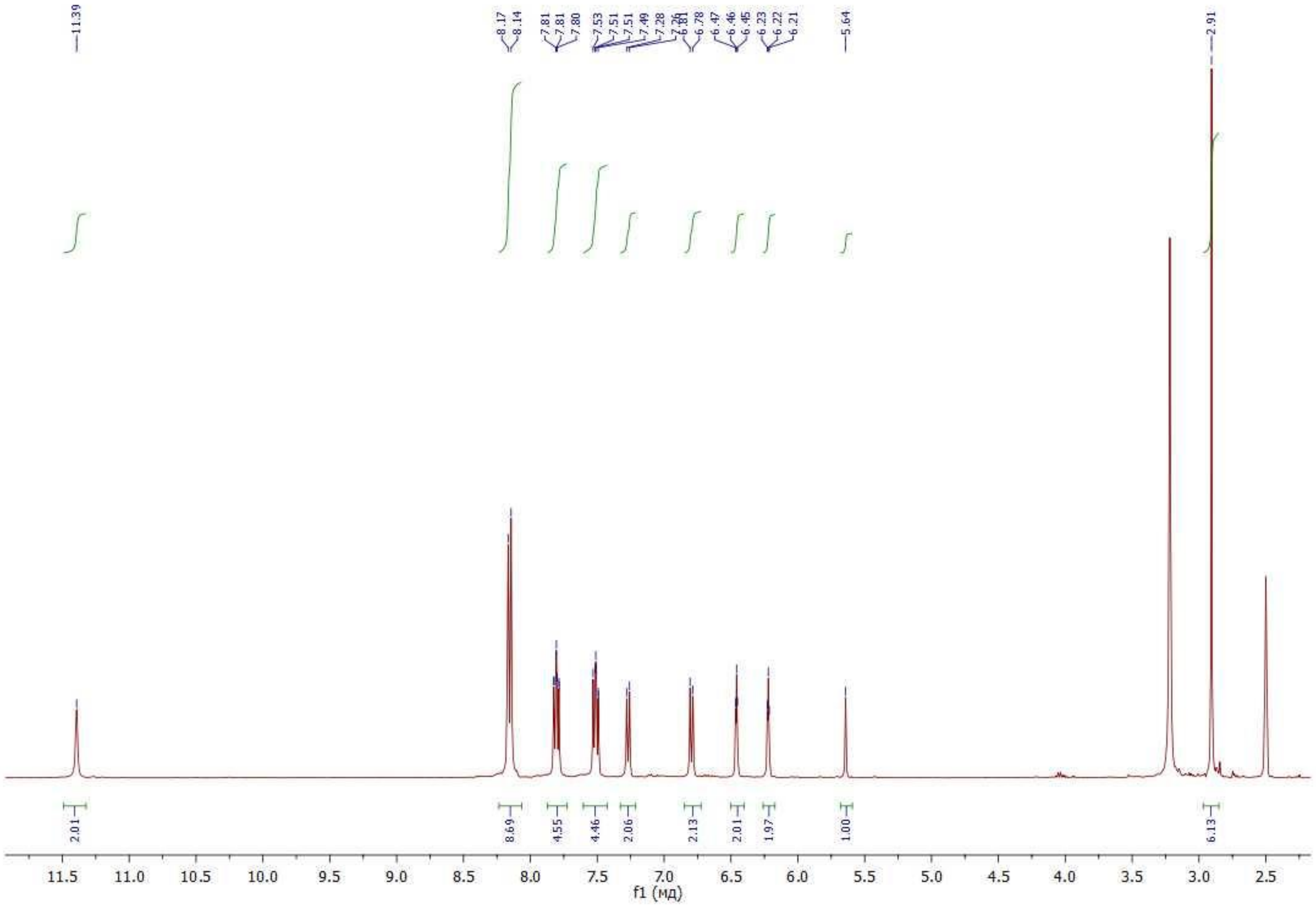


**Figure S13.** $^{1}$**H NMR Spectrum of 5g**

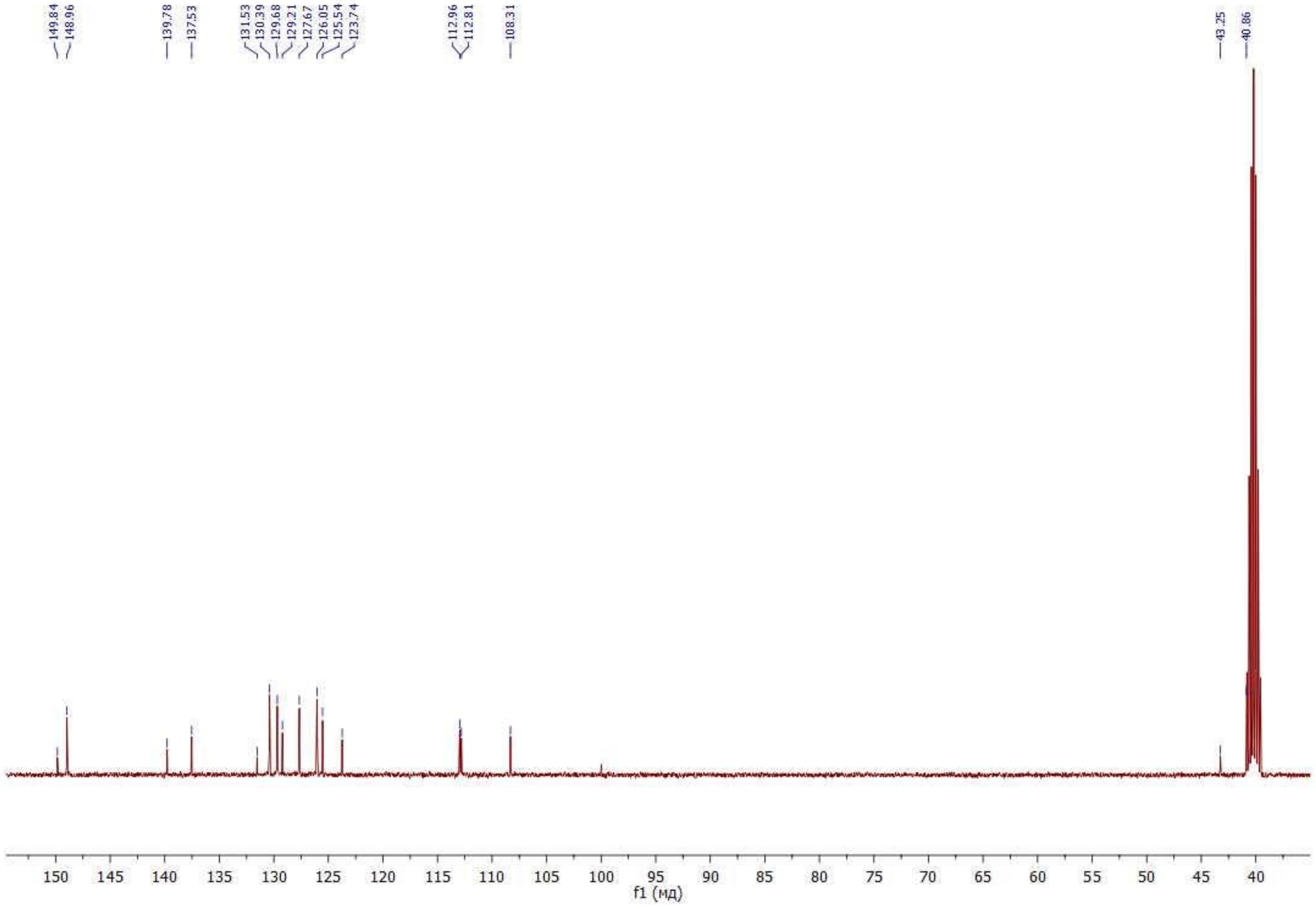


**Figure S14.** **$^{13}$C NMR Spectrum of 5g**

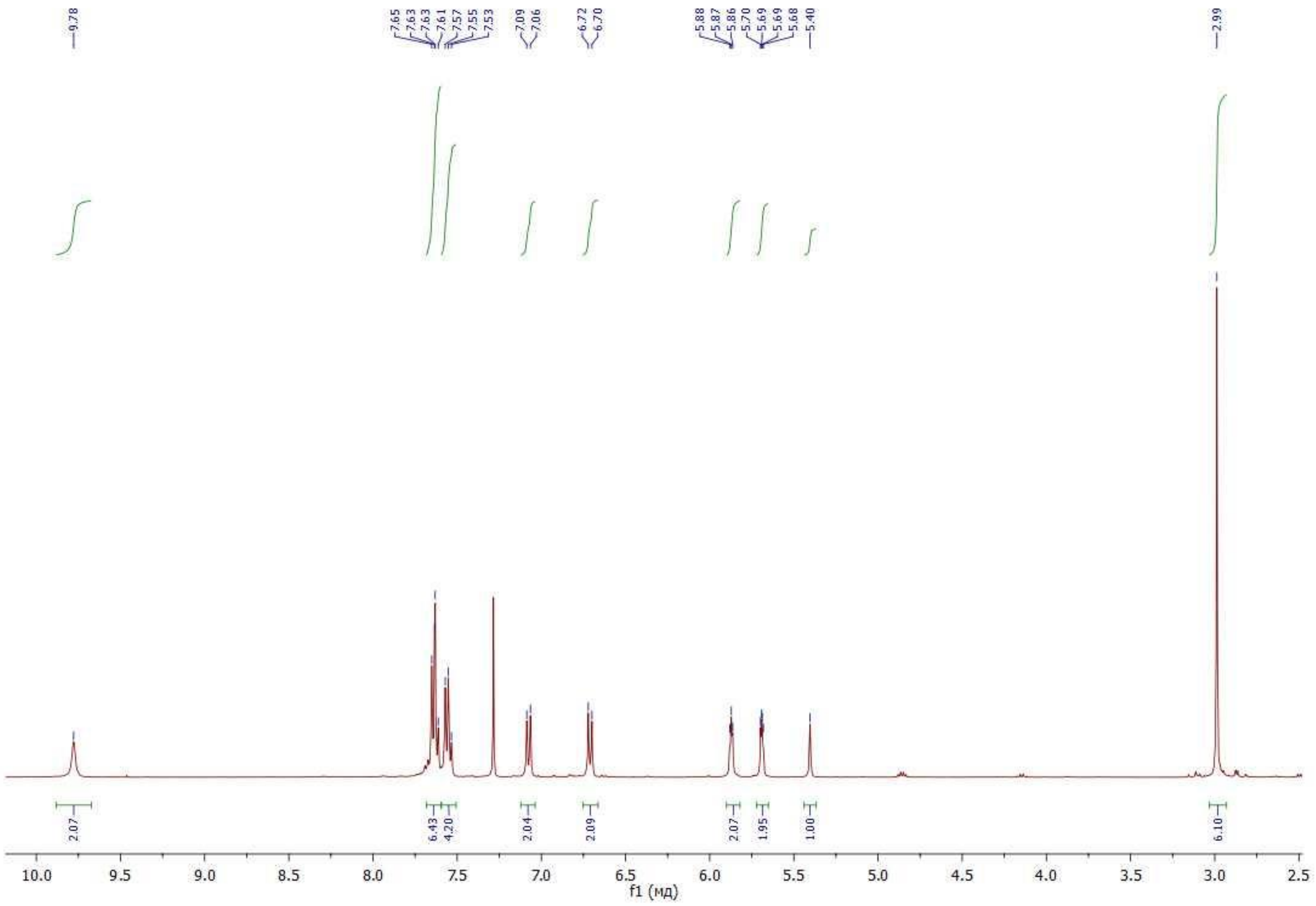


**Figure S15. $^1$H NMR Spectrum of 5h**

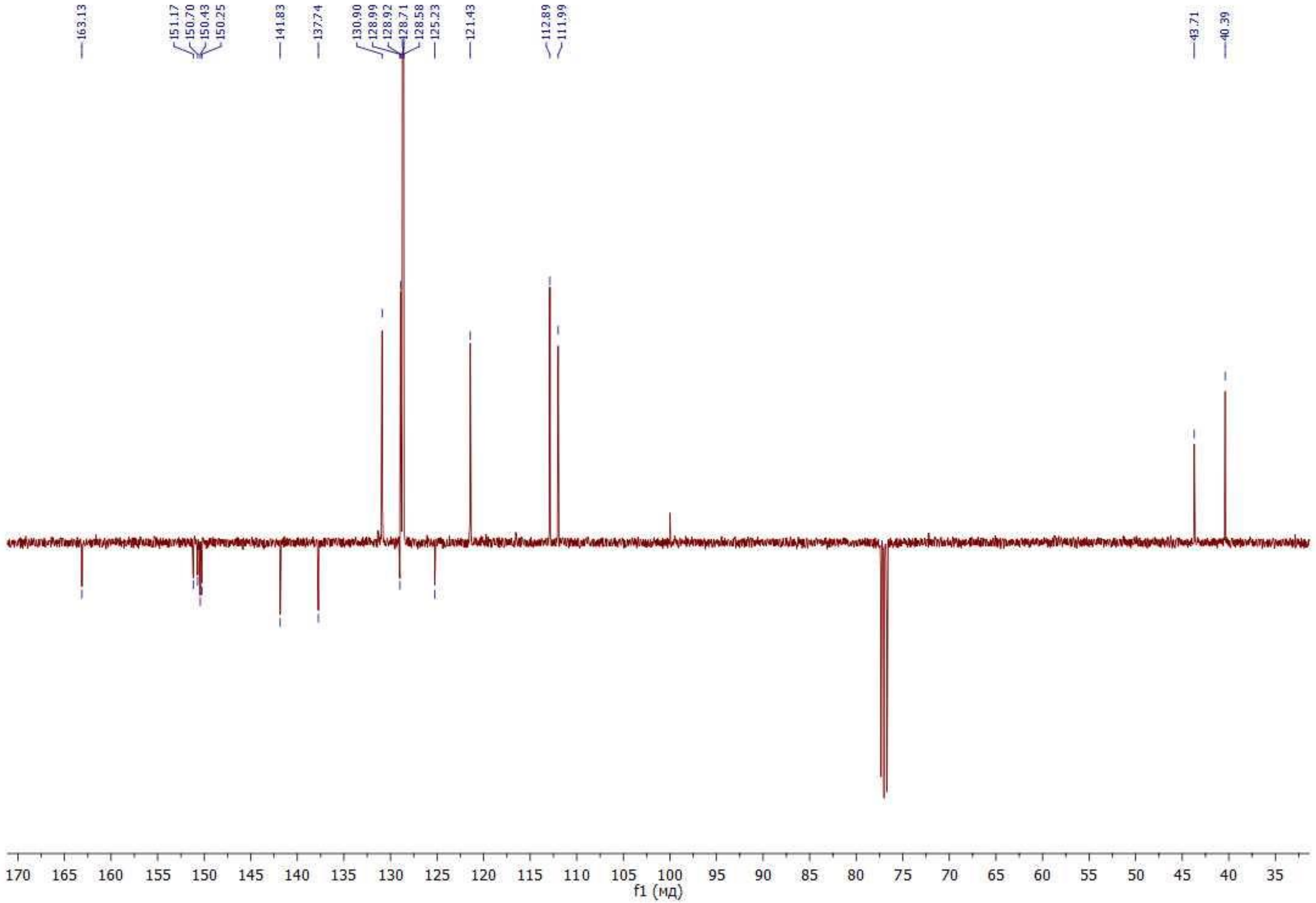


**Figure S16.** $^{13}C$ **NMR Spectrum of 5h**

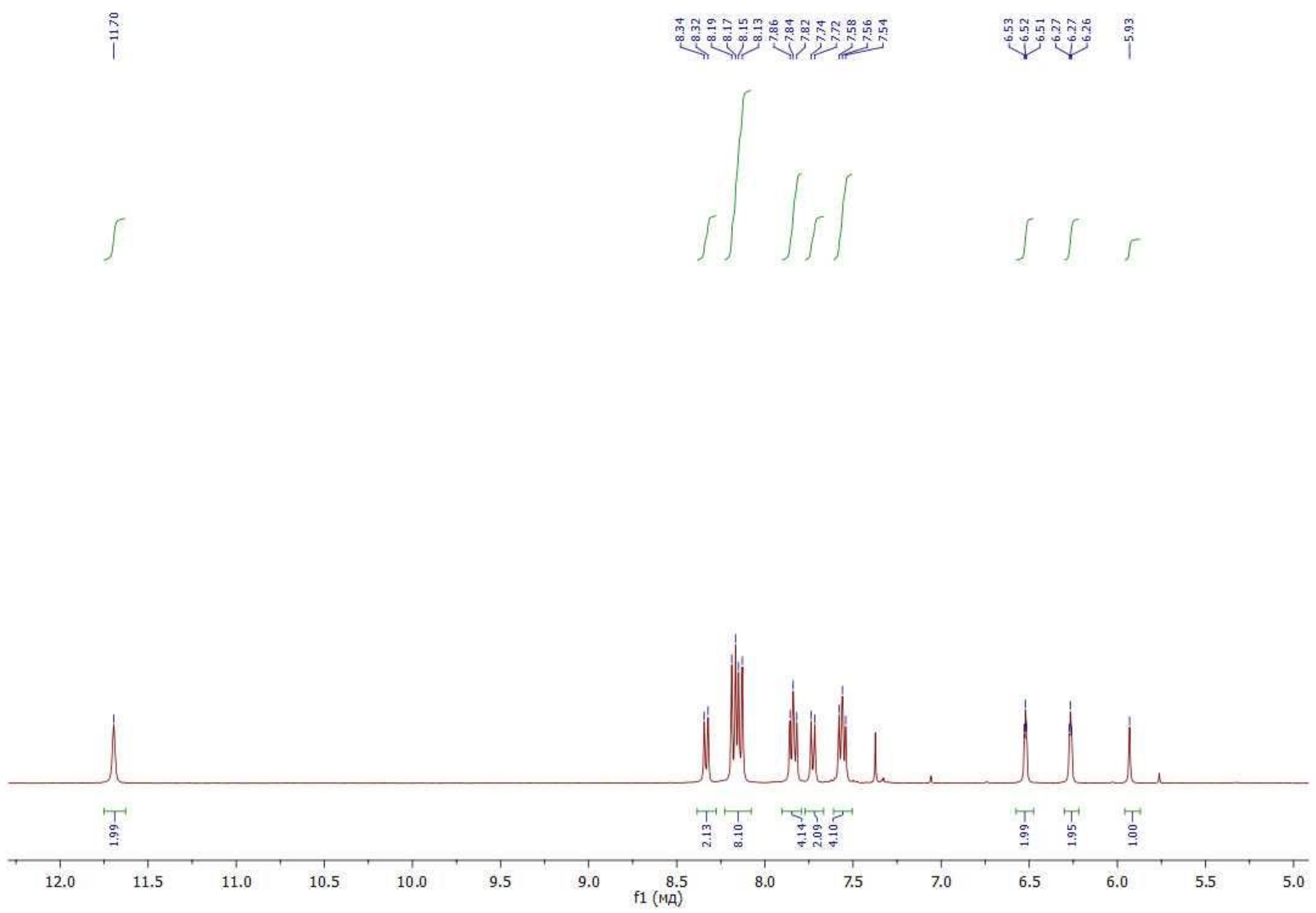


**Figure S17.** $^{1}H$ **NMR Spectrum of 5i**

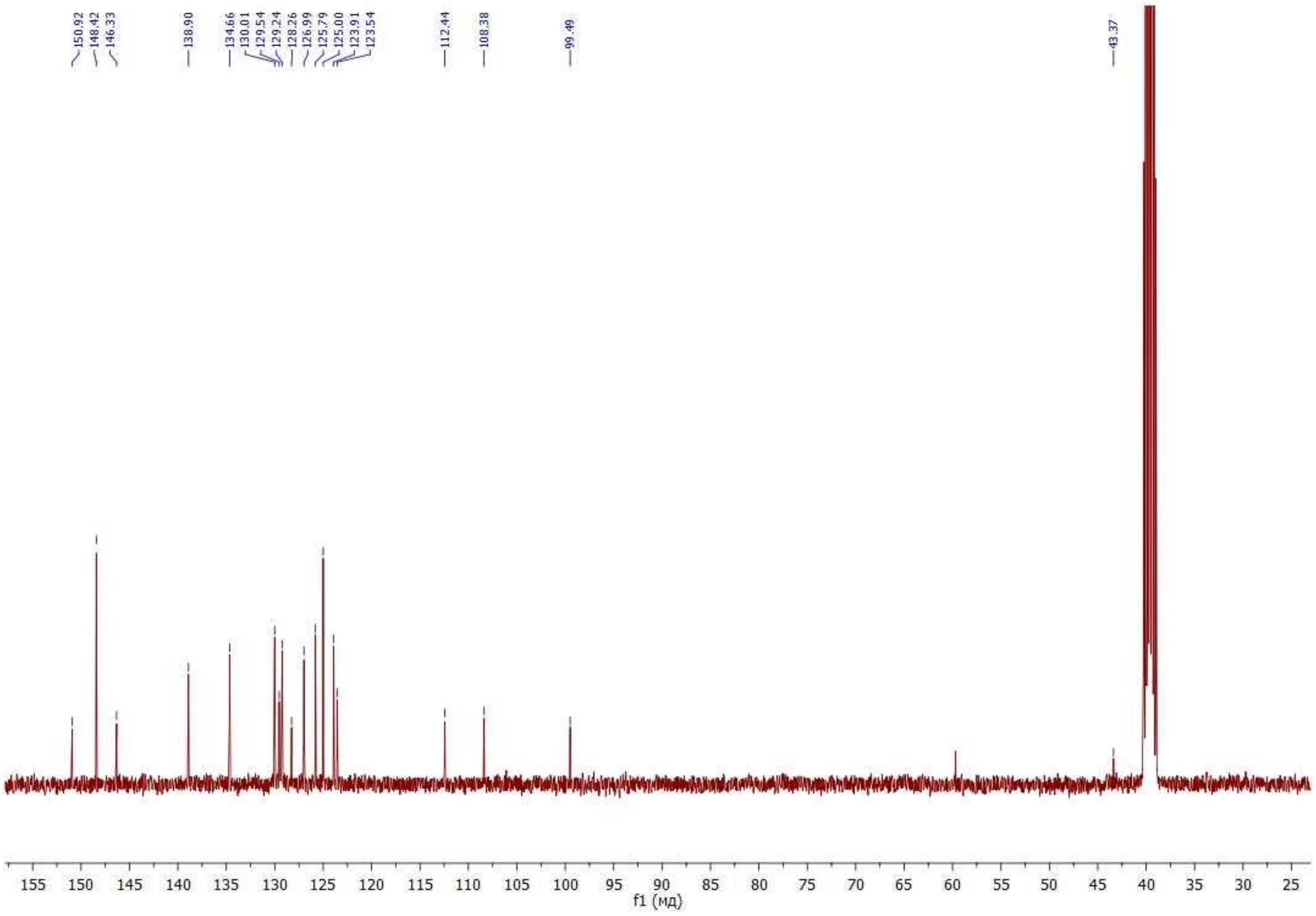


**Figure S18.** $^{13}C$ **NMR Spectrum of 5i**

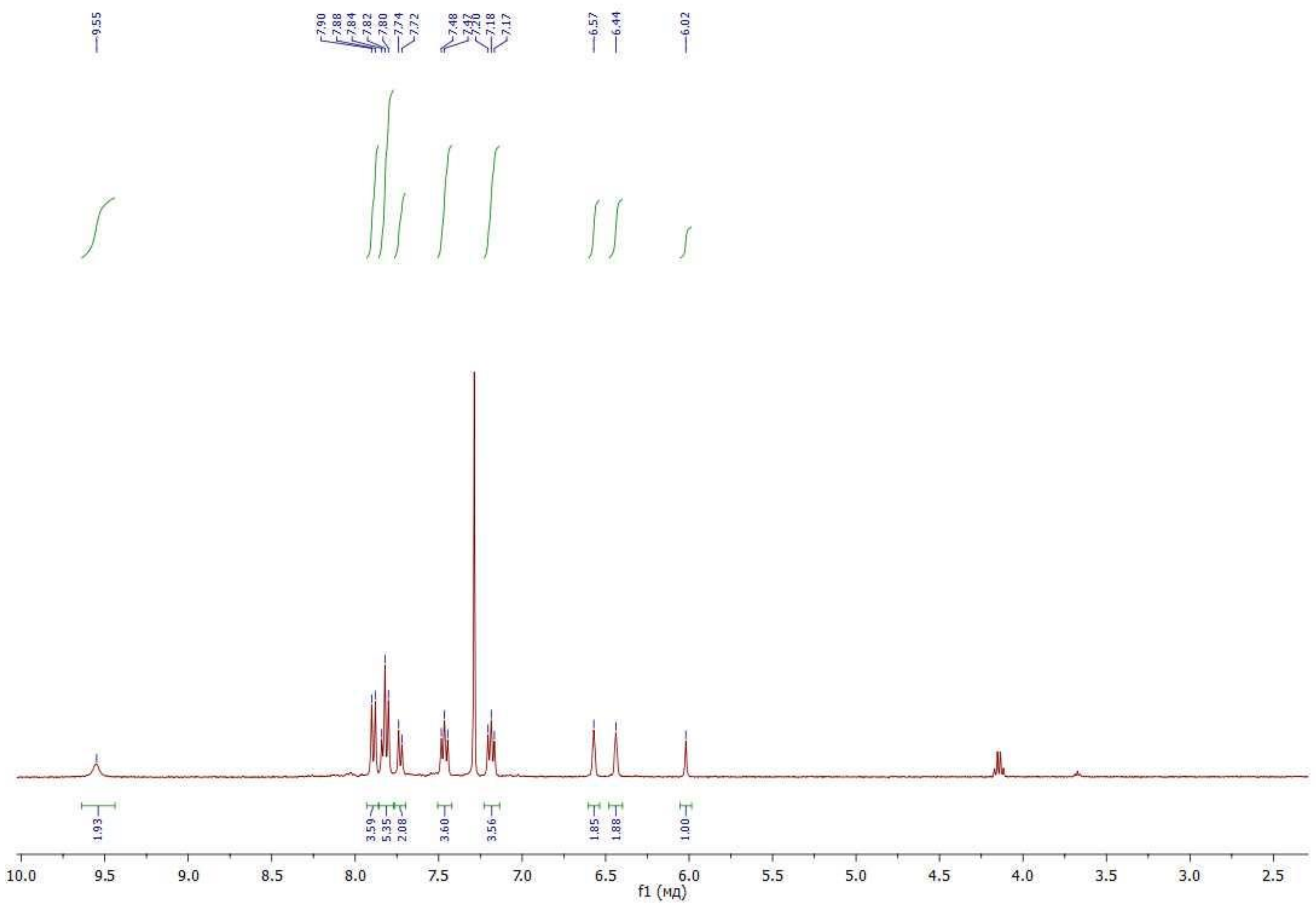


**Figure S19. $^1$H NMR Spectrum of 5j**

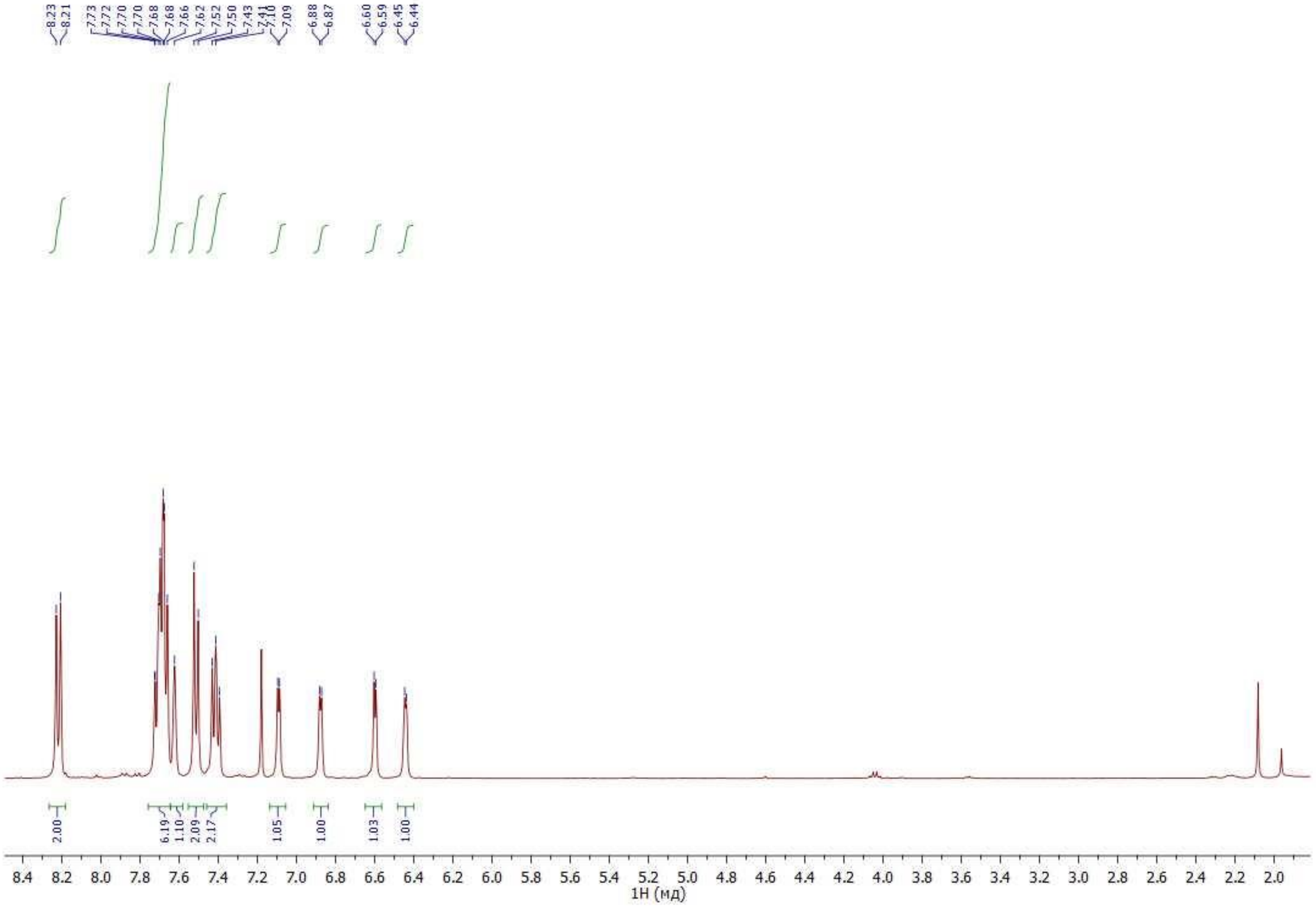


**Figure S20.** [1]**H NMR Spectrum of 6a**

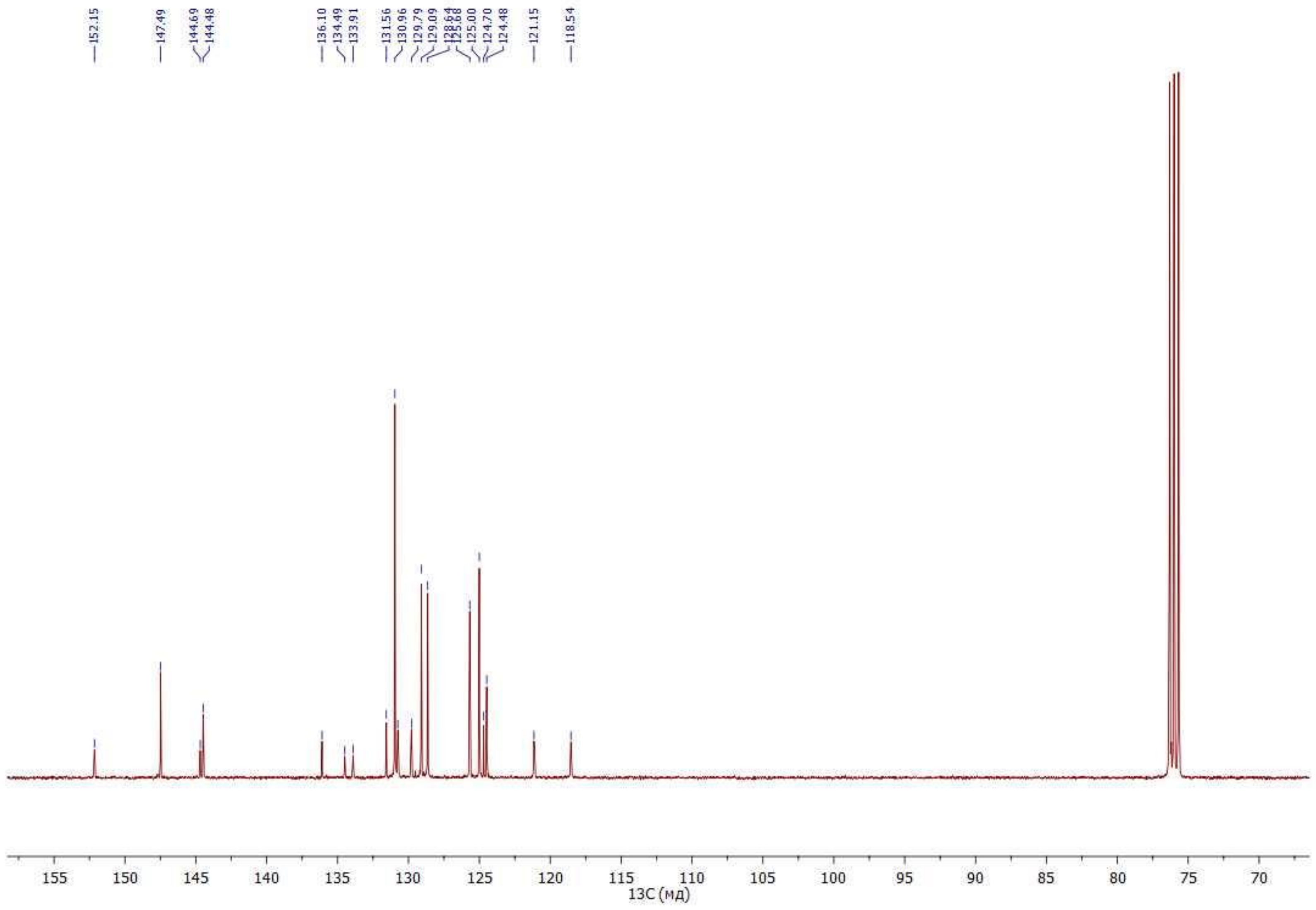


**Figure S21.** $^{13}C$ **NMR Spectrum of 6a**

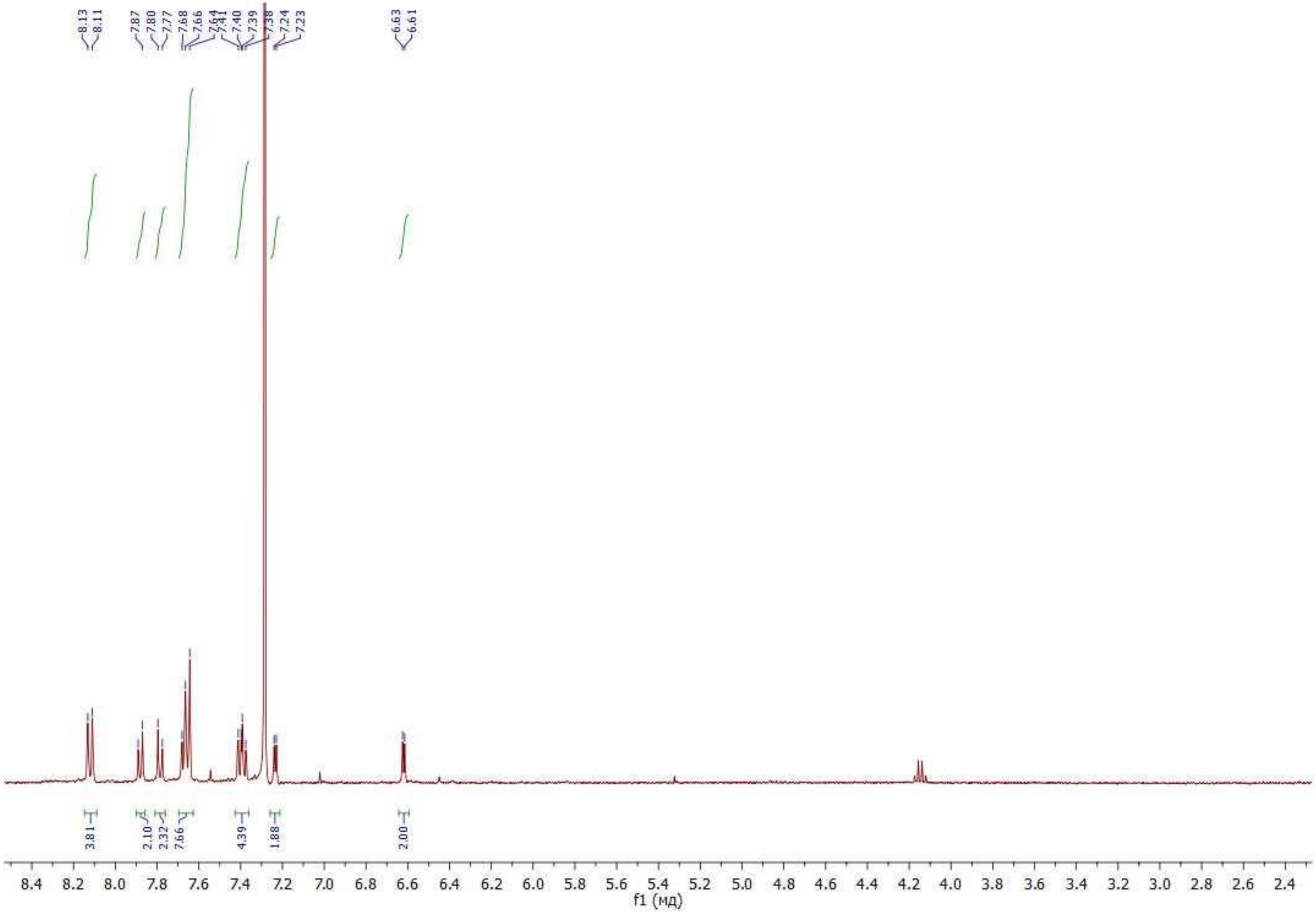


**Figure S22. ¹H NMR Spectrum of 6b**

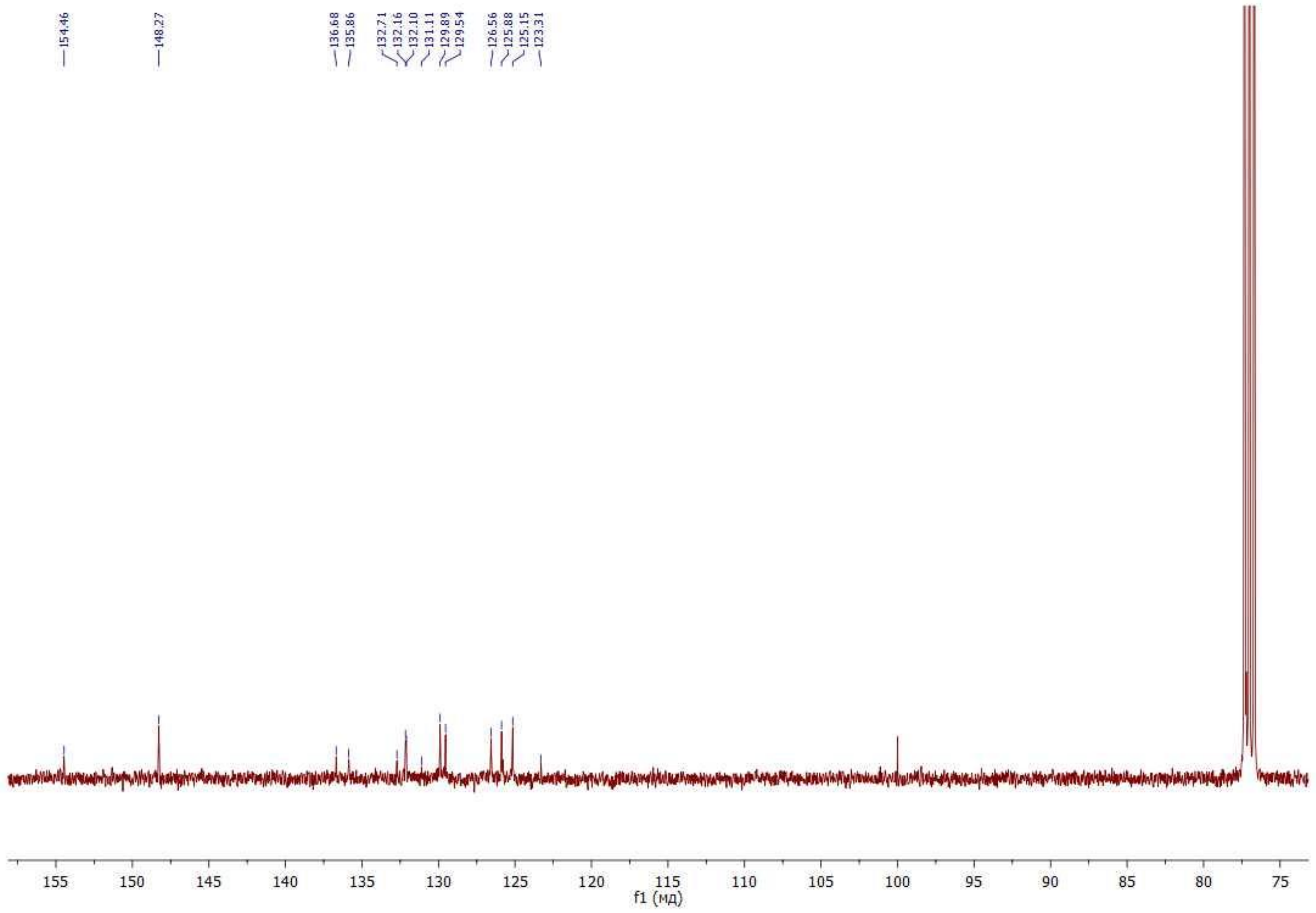


**Figure S23.** $^{13}C$ **NMR Spectrum of 6b**

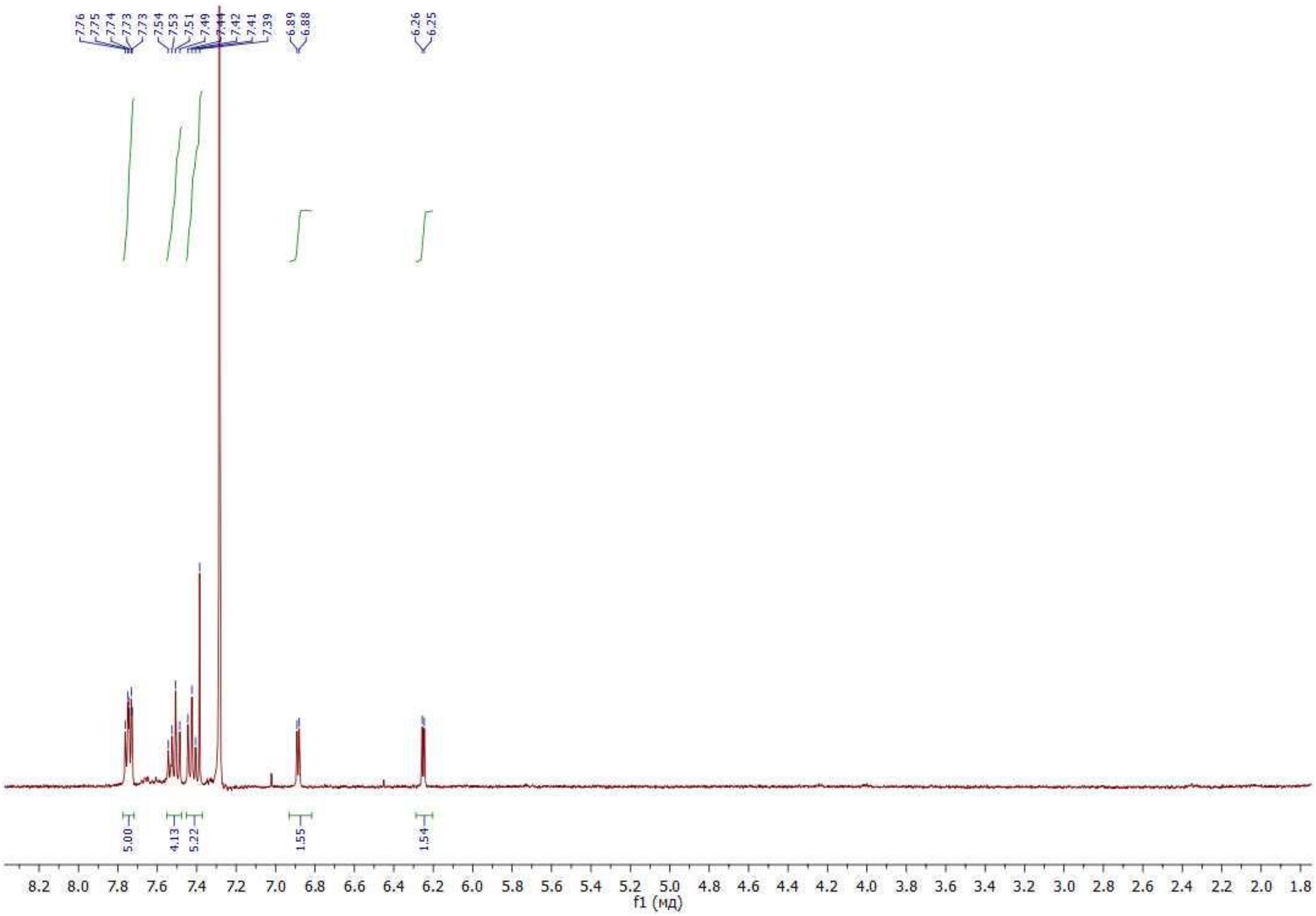


**Figure S24. $^1$H NMR Spectrum of 6c**

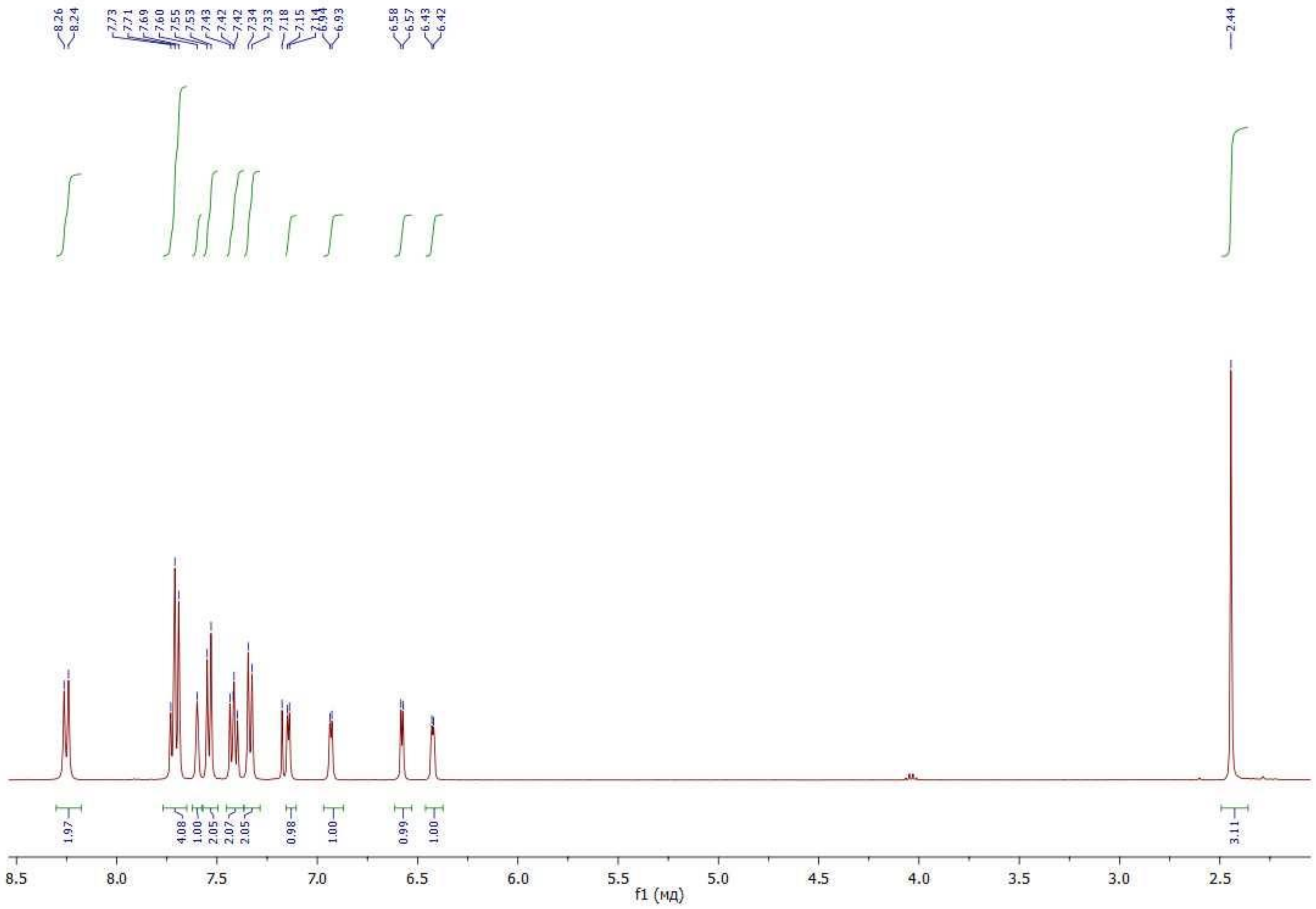


**Figure S25.** $^{1}H$ **NMR Spectrum of 6d**

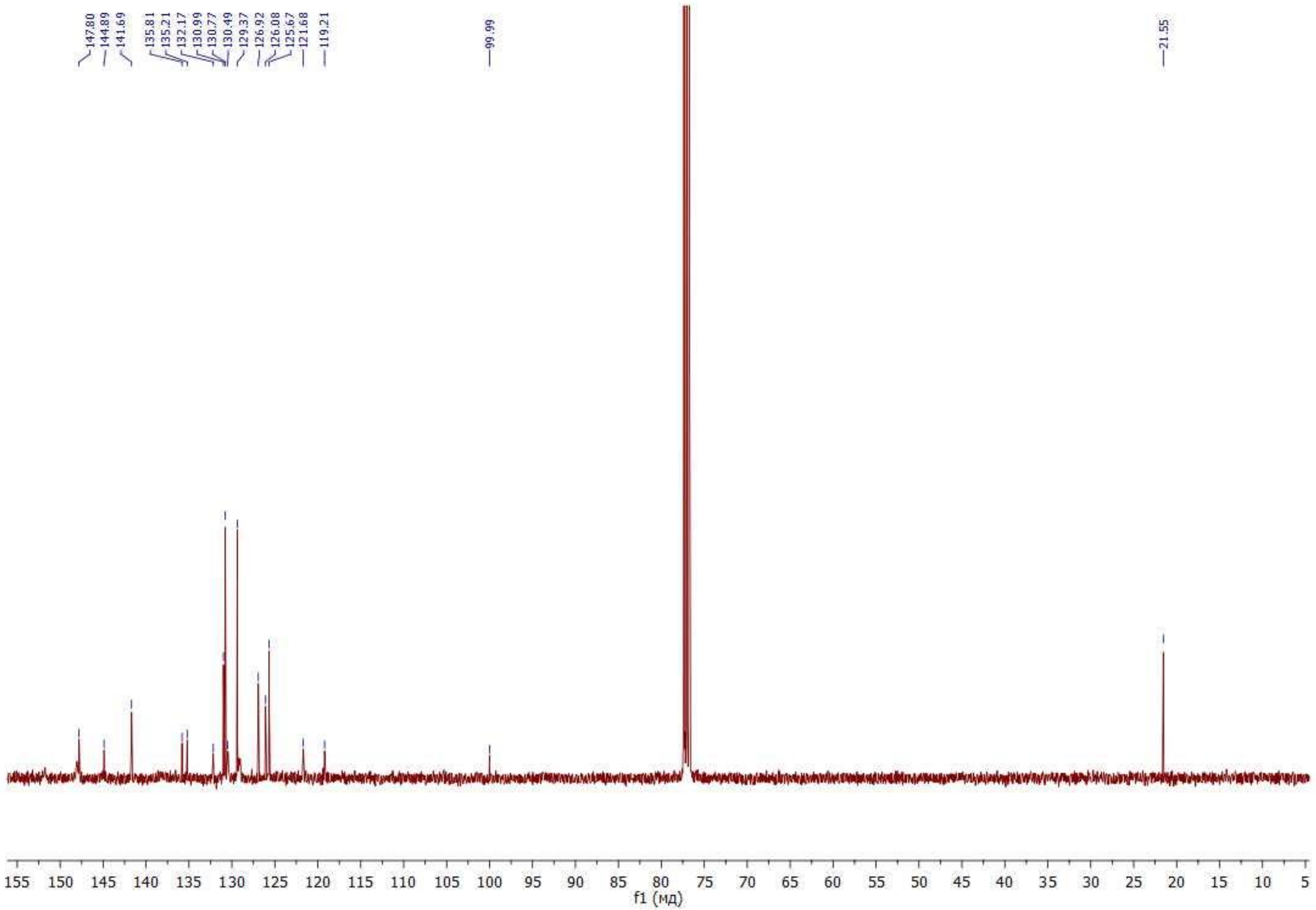


**Figure S26.** $^{13}C$ **NMR Spectrum of 6d**

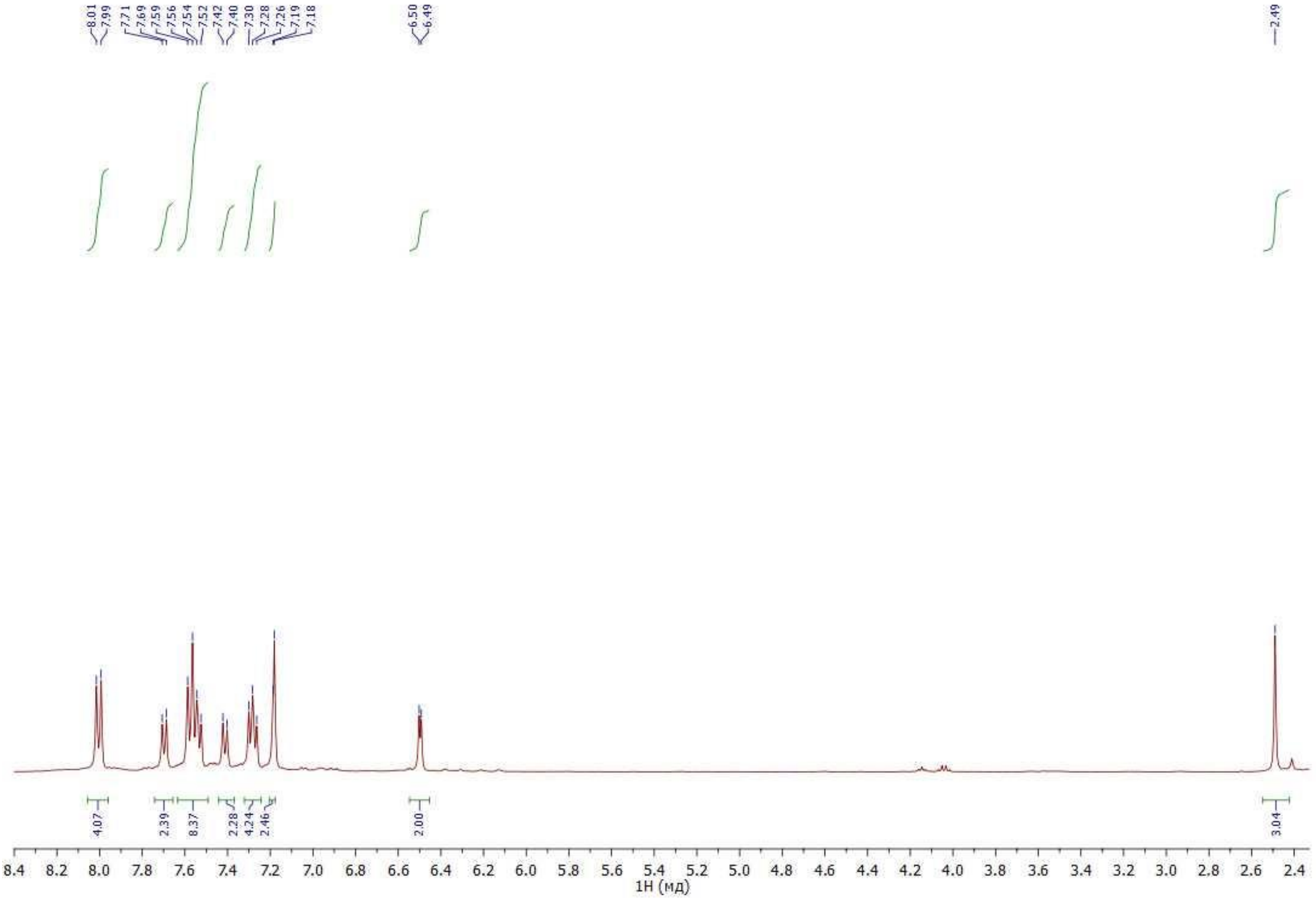


**Figure S27. $^{1}$H NMR Spectrum of 6e**

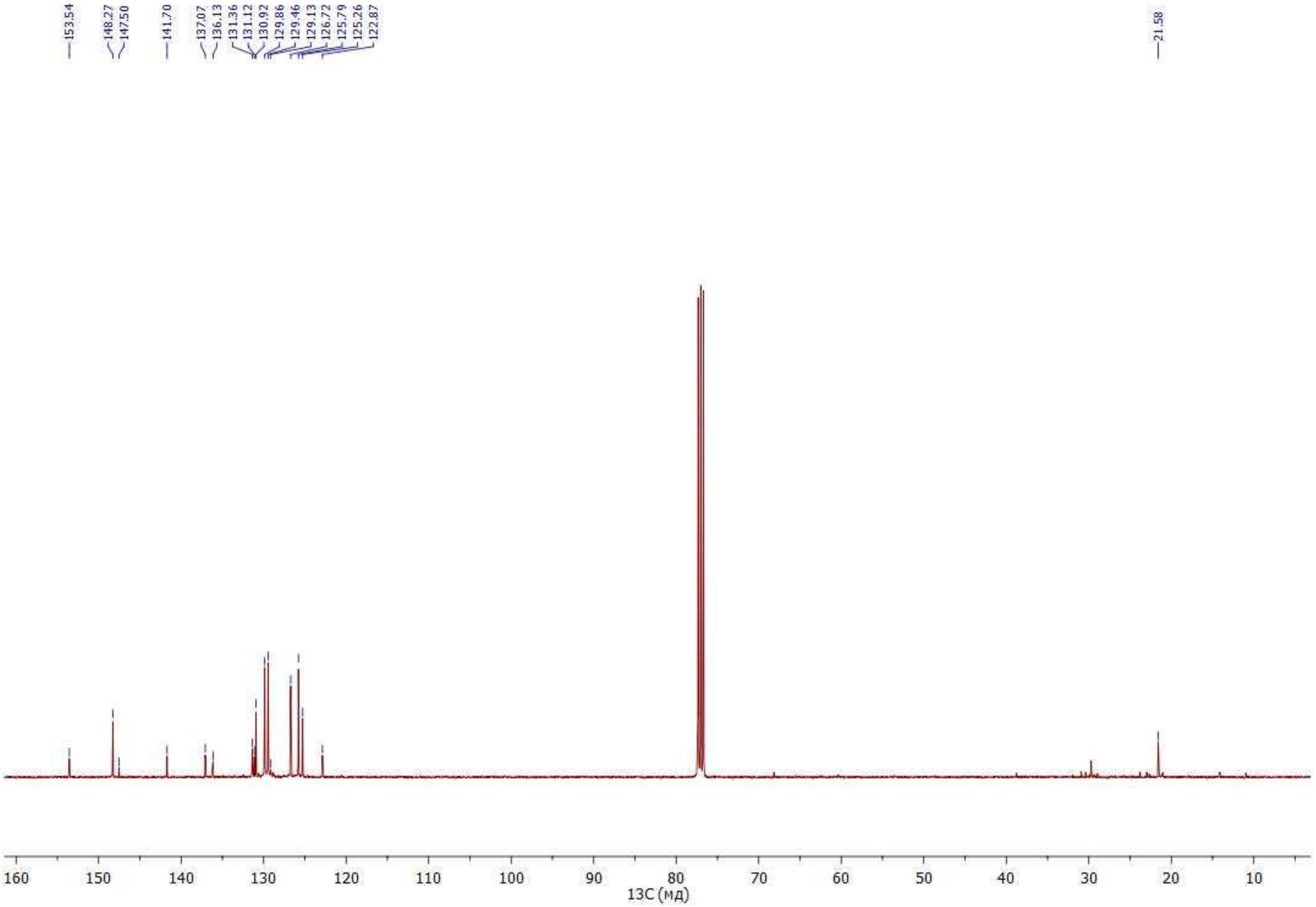


**Figure S28.** **$^{13}$C NMR Spectrum of 6e**

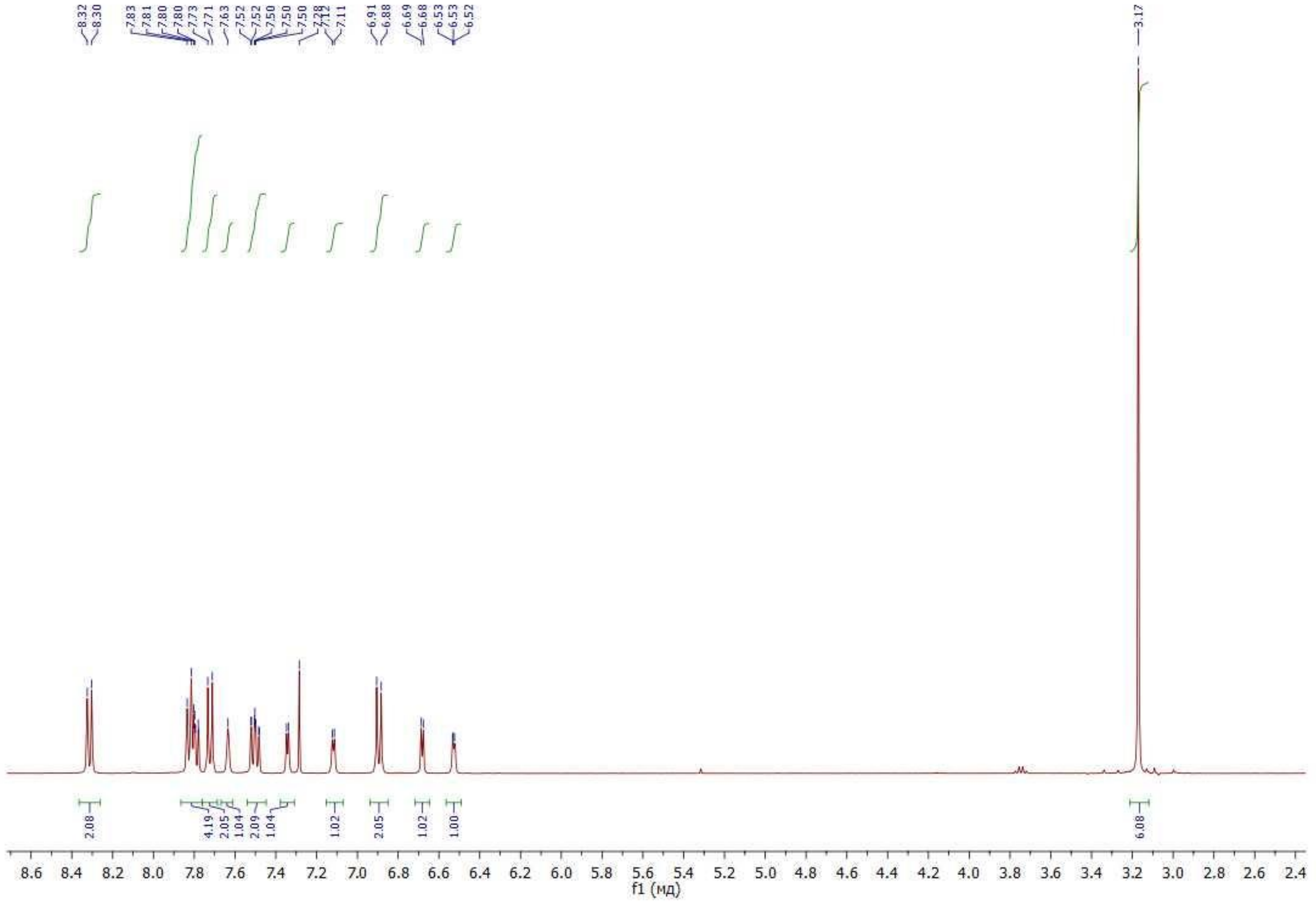


**Figure S29. ¹H NMR Spectrum of 6f**

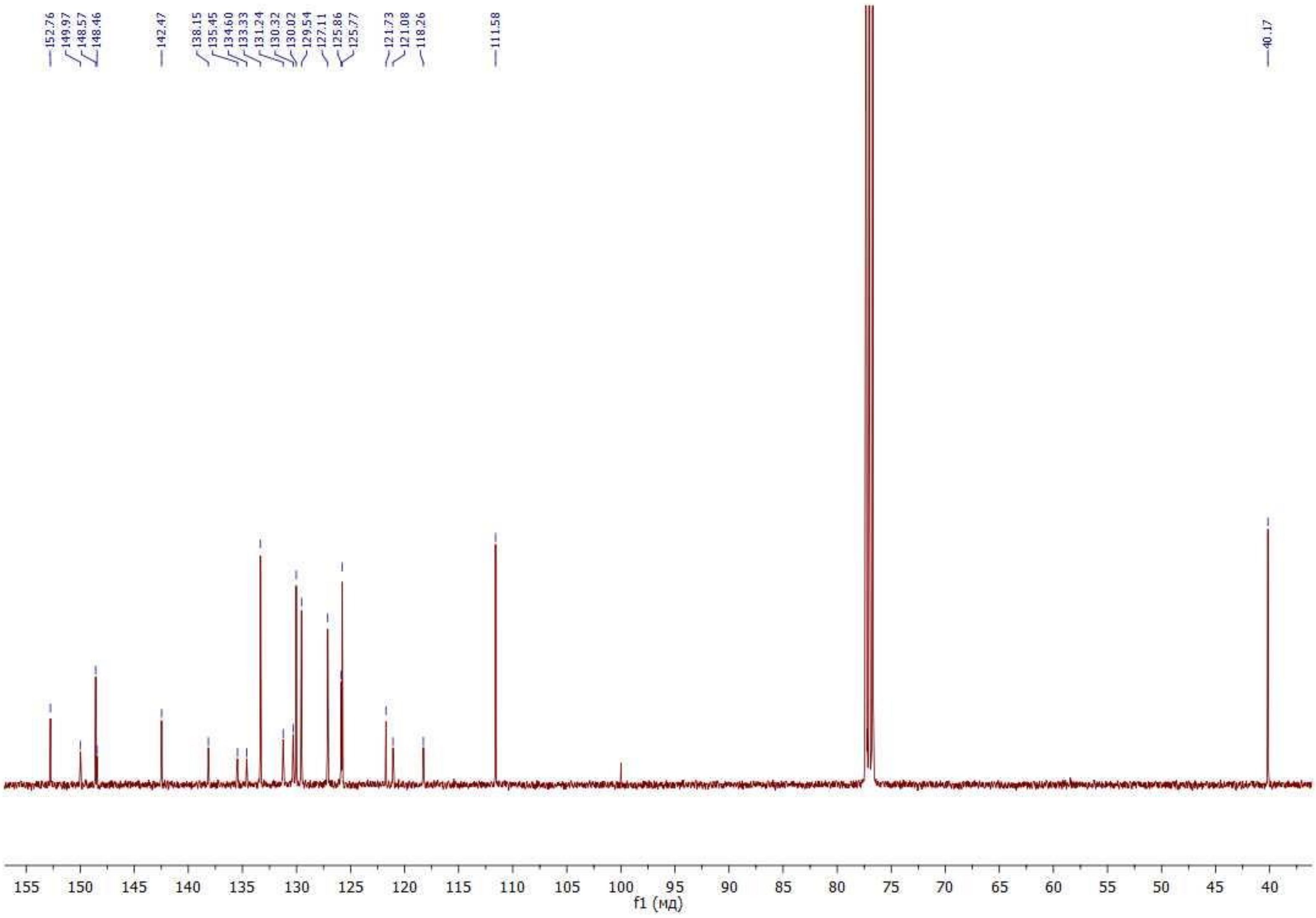


**Figure S30.** $^{13}C$ **NMR Spectrum of 6f**

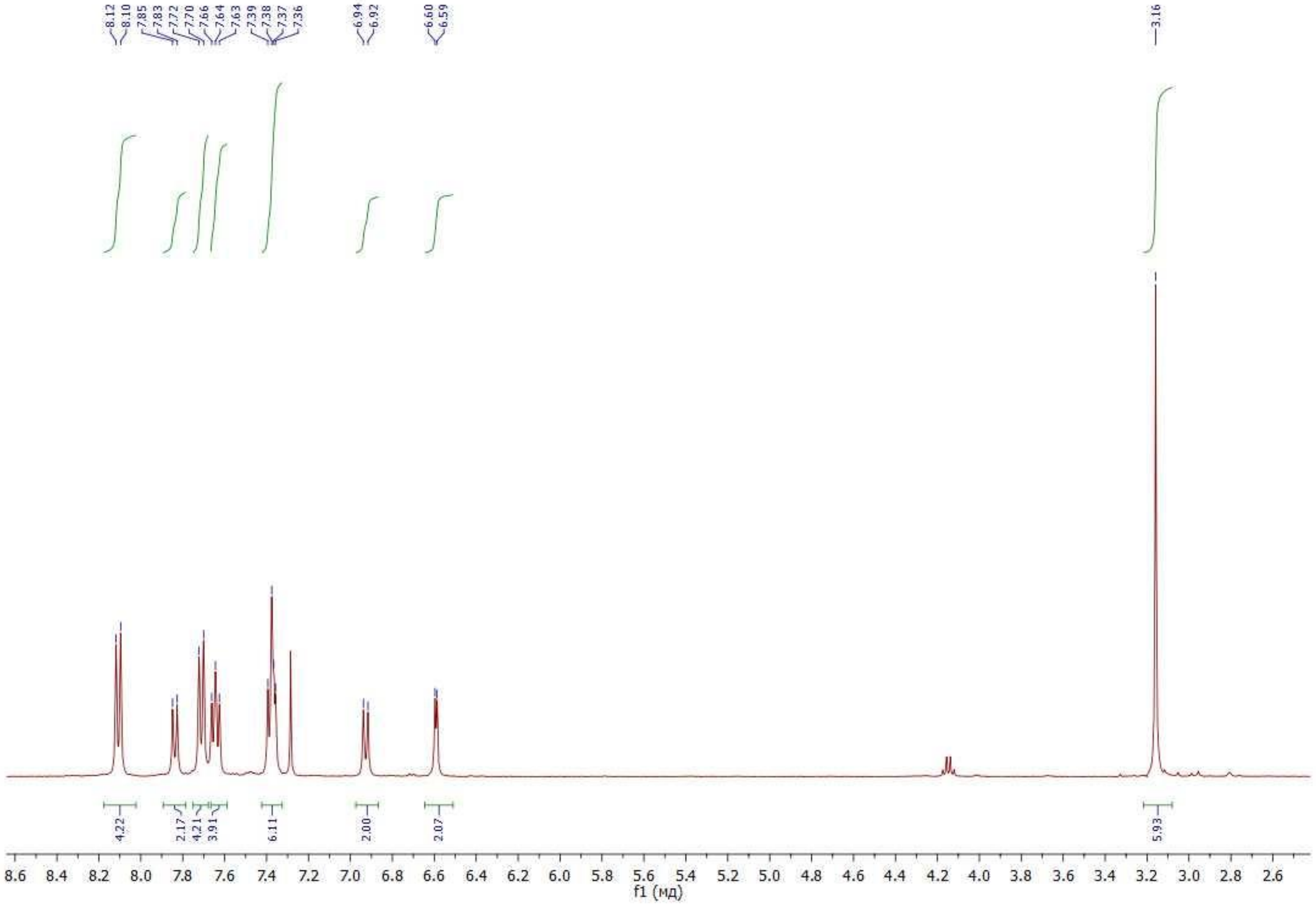


**Figure S31.** $^1$**H NMR Spectrum of 6g**

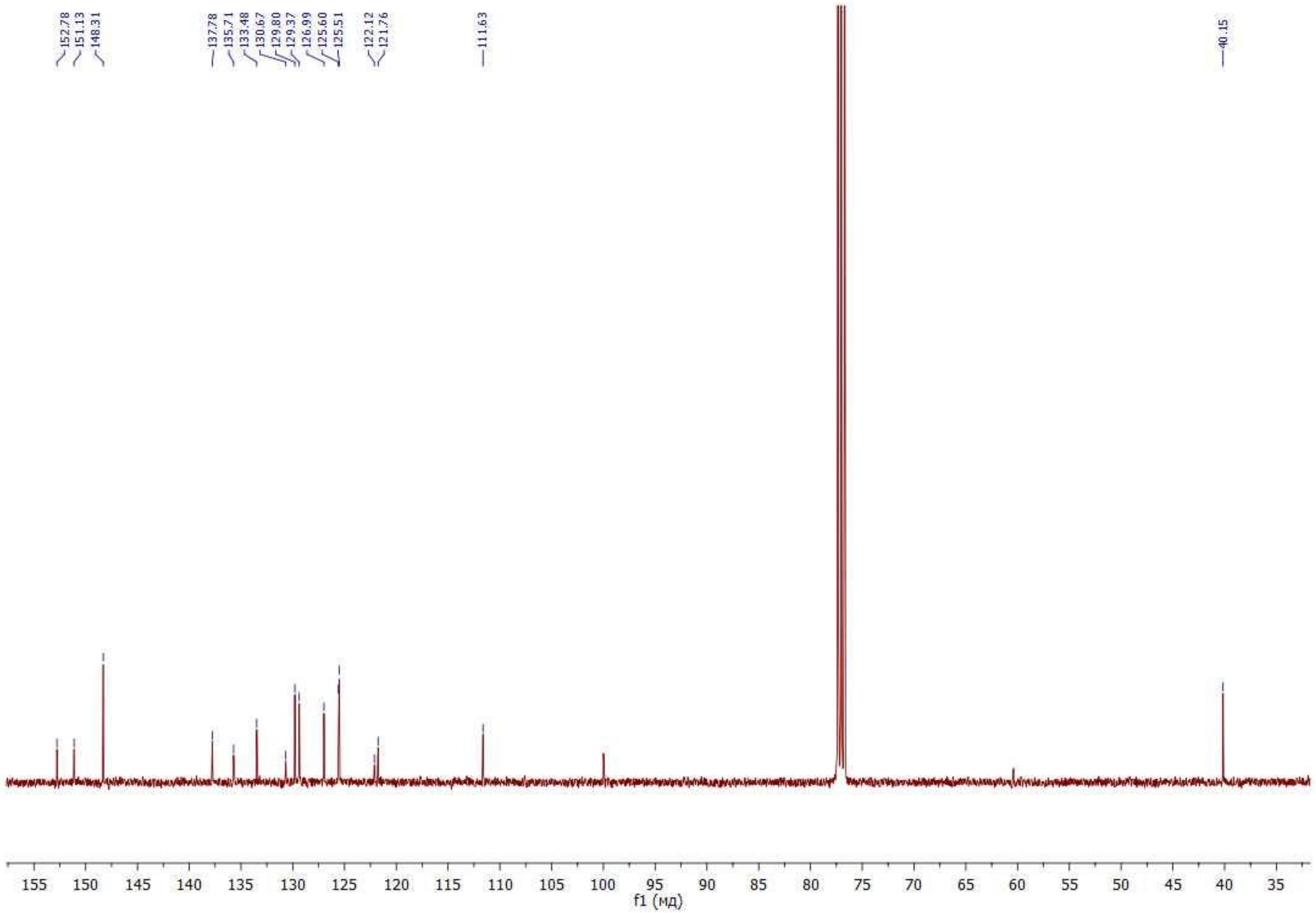


**Figure S32.** $^{13}C$ **NMR Spectrum of 6g**

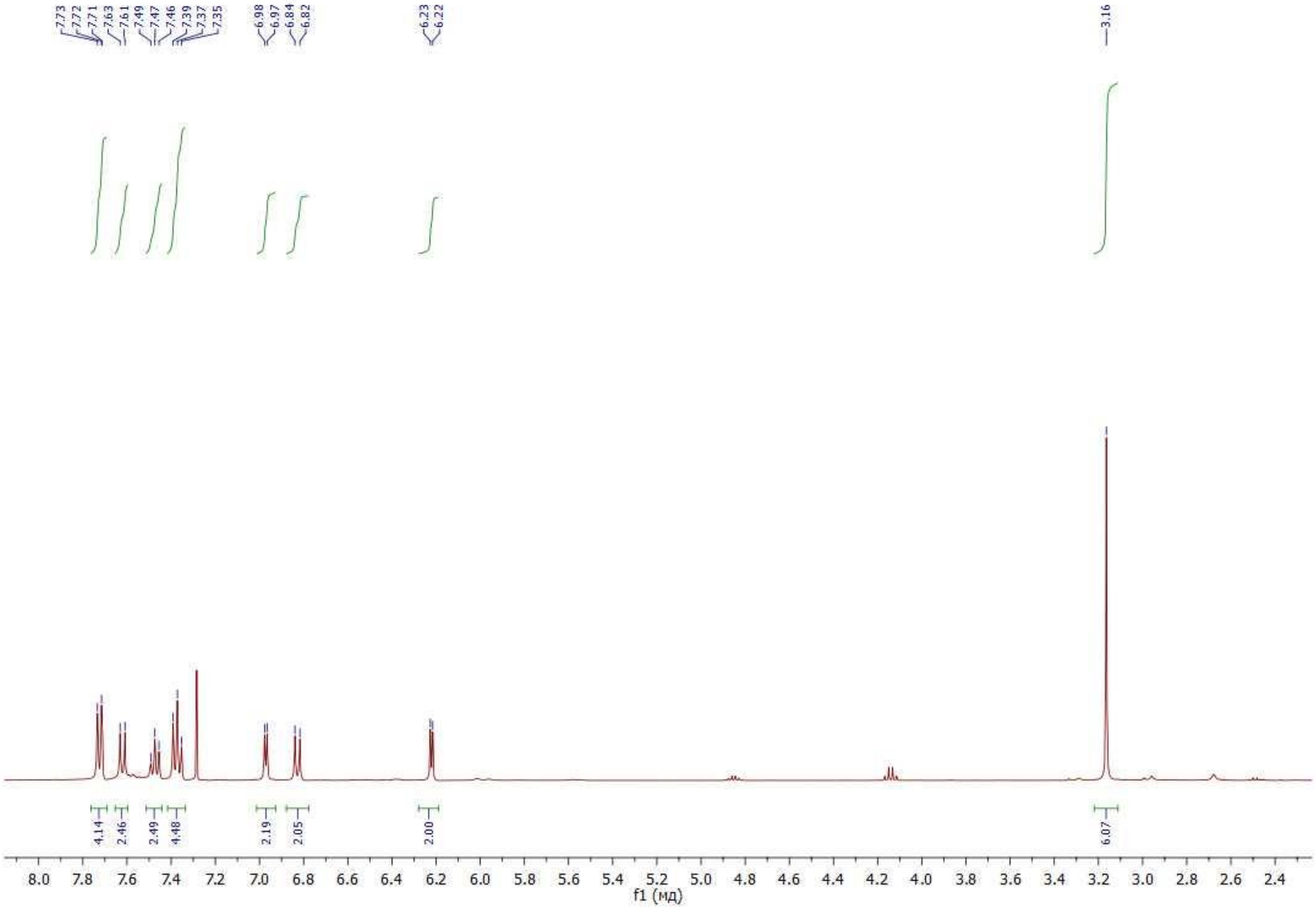


**Figure S33. $^{1}$H NMR Spectrum of 6h**

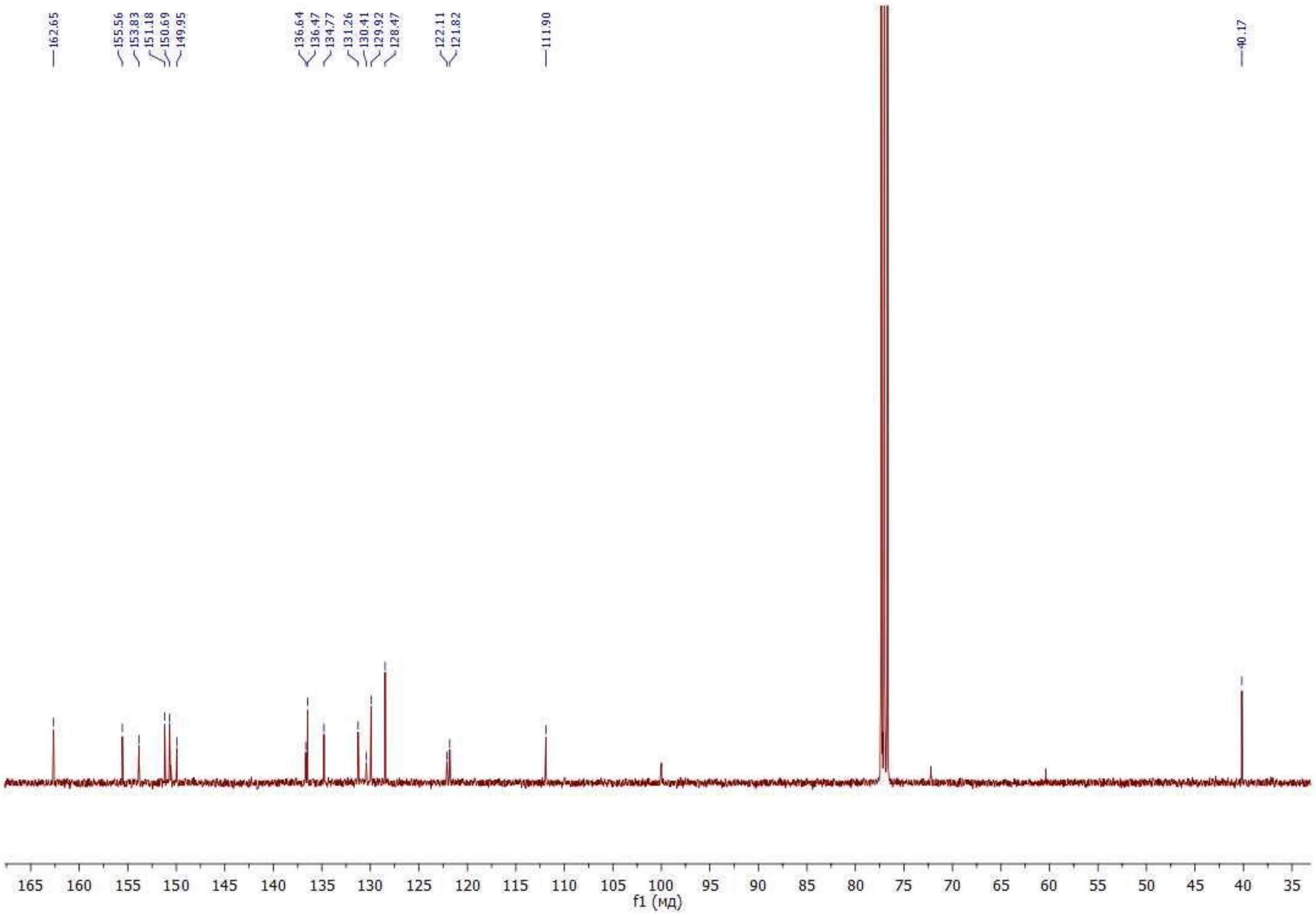


**Figure S34.** **$^{13}$C NMR Spectrum of 6h**

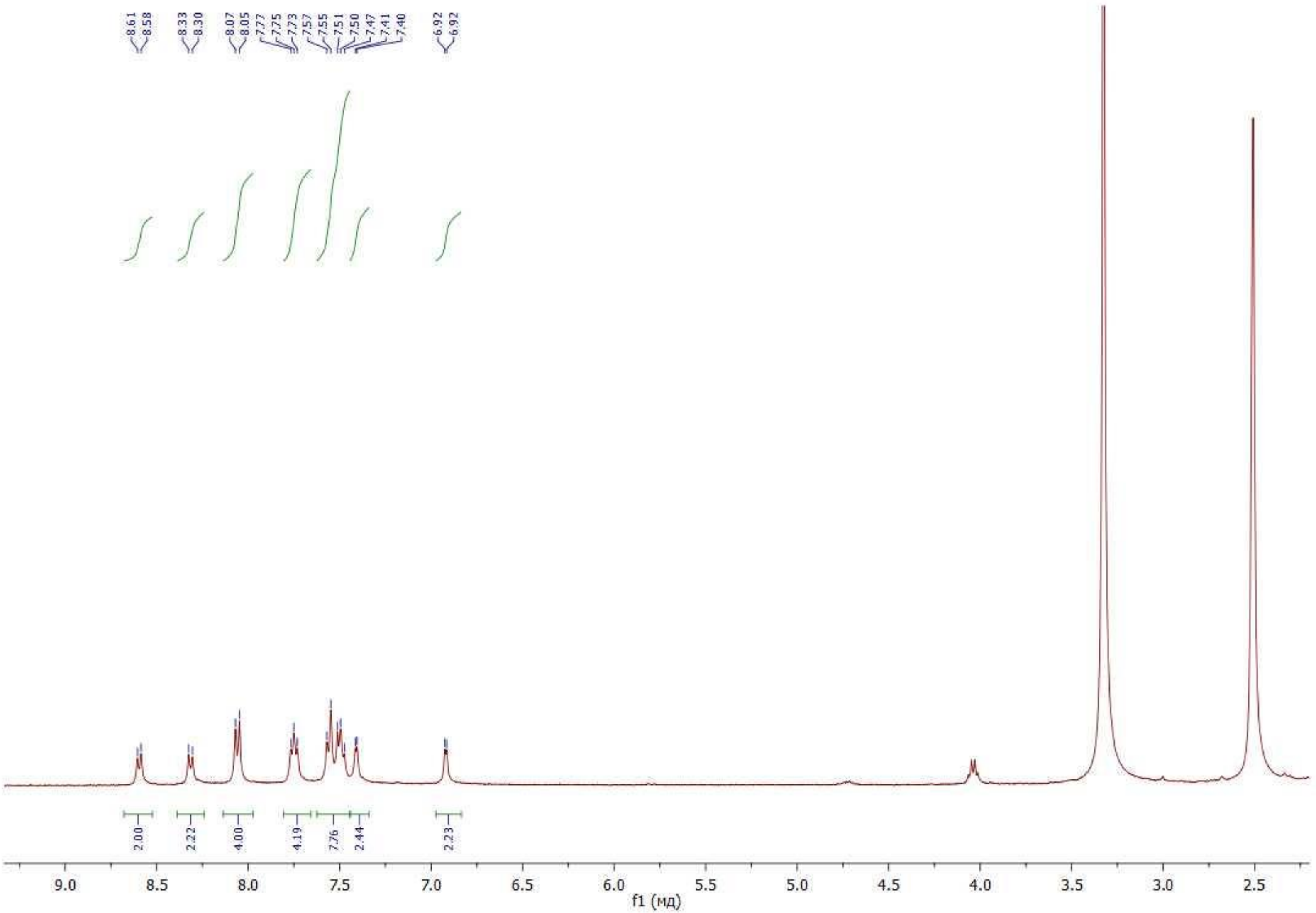


**Figure S35.** **$^{1}$H NMR Spectrum of 6i**

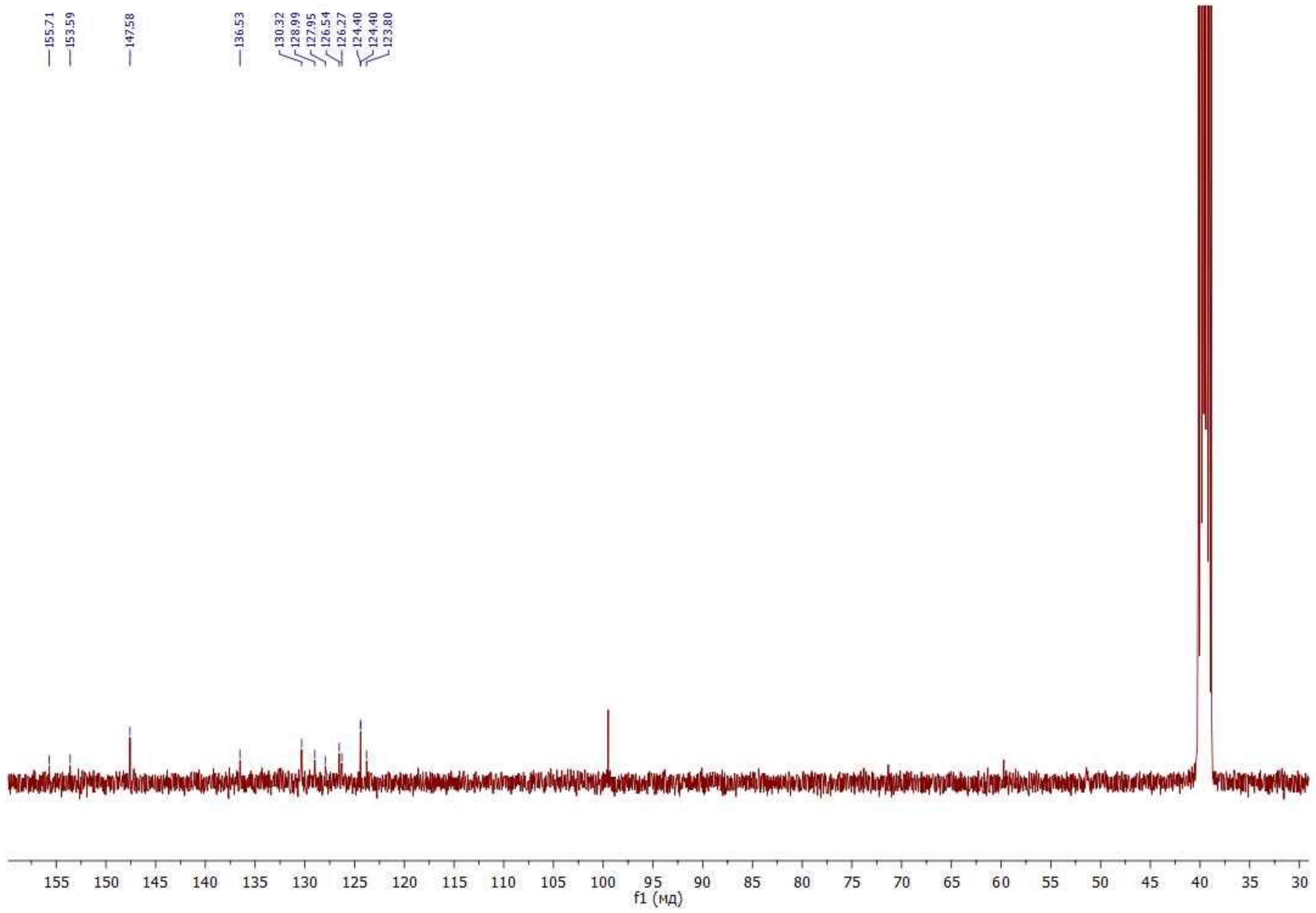


**Figure S36.** $^{13}C$ **NMR Spectrum of 6i**

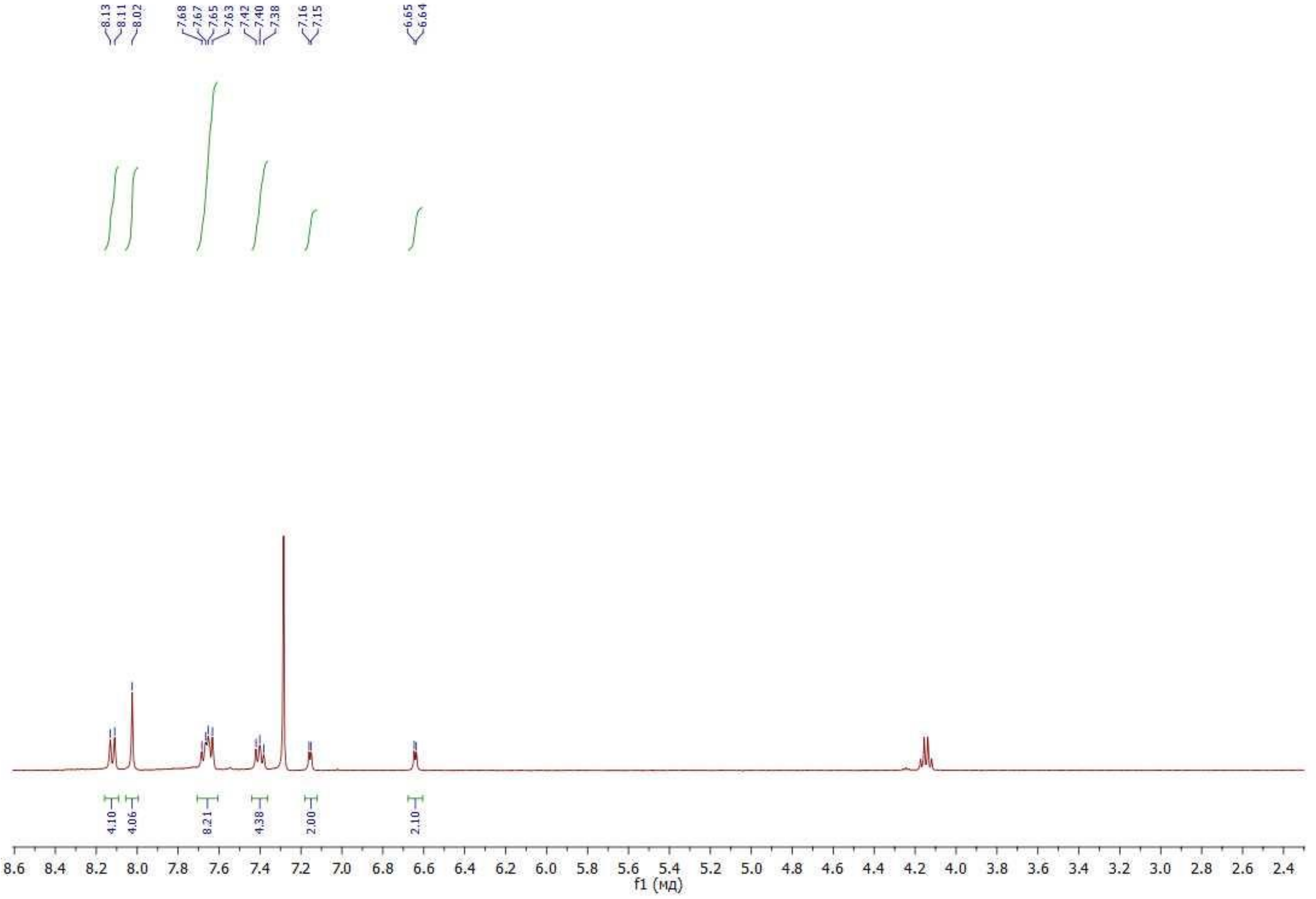


**Figure S37. $^{1}$H NMR Spectrum of 6j**
